# A Versatile Protein Scaffold Engineered for the Hierarchical Assembly of Robust and Highly Active Enzymes

**DOI:** 10.1002/advs.202500405

**Published:** 2025-02-22

**Authors:** Yiwei Meng, Lukasz Peplowski, Tong Wu, Heng Gong, Ran Gu, Laichuang Han, Yuanyuan Xia, Zhongmei Liu, Zhemin Zhou, Zhongyi Cheng

**Affiliations:** ^1^ Key Laboratory of Industrial Biotechnology (Ministry of Education) School of Biotechnology Jiangnan University Wuxi Jiangsu China; ^2^ Institute of Physics Faculty of Physics Astronomy and Informatics Nicolaus Copernicus University in Torun Grudziadzka 5 Torun 87–100 Poland; ^3^ Jiangnan University (Rugao) Food Biotechnology Research Institute Rugao Jiangsu China

**Keywords:** dual cascades, enhanced guest enzyme performances, protein scaffolds, self‐assembly

## Abstract

Scaffold proteins play immense roles in bringing enzymes together to enhance their properties. However, the direct fusion of scaffold with bulky guest enzymes may disrupt the assembly process or diminish catalytic efficiency. Most self‐assembling protein scaffolds are engineered to form structures beforehand, and then carry guest proteins via different conjugation strategies in vitro. Here, a robust self‐assembling scaffold is presented, engineered from *Methanococcus jannaschii* using disulfide bonds, which efficiently assembles bulky enzymes into higher‐order helices without additional chemistry or bio‐conjugation in vitro. When fused directly with monomeric Endo‐1,4‐beta‐xylanase A, the catalytic efficiency of the guest enzyme increased by 2.5 times with enhanced thermostability. Additionally, integrating the scaffold with the multimeric metalloenzyme nitrile hydratase overcame the typical stability‐activity trade‐off of such industrial enzyme, yielding three‐fold higher activity and 28‐fold higher thermostability. Structural analyses suggest that the artificially made helical twist structures create new interface interactions and provide a concentration of active sites of guest enzymes. Further fusion of fluorescent protein pairs with the scaffold exhibited a 12‐fold higher FRET efficiency, suggesting its potential for dual‐enzyme cascade applications. Overall, this study showcases a simple yet powerful protein scaffold that organizes guest enzymes into hierarchical structures with enhanced catalytic performance.

## Graphical abstract

This study introduces a self‐assembling protein scaffold engineered from *Methanococcus jannaschii* via disulfide bonds, enabling efficient in vivo assembly of bulky enzymes into helical structures without conjugation. Direct fusion increased the activity and thermostability of both monomeric and multimeric guest enzymes. Structural analysis revealed helical interfaces concentrate active sites. This scaffold simplifies hierarchical enzyme organization for enhanced catalysis.

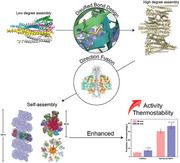

## 1 Introduction

Naturally occurring enzymes exhibit diverse catalytic abilities due to their flexible three‐dimensional structures, yet their inherent instability hinders their industrial application.^[^ ^1^ ^]^ Various strategies have been devised to fine‐tune catalytic functions and enhance enzyme robustness, including: i) altering enzyme structures via direct evolution or rational design; ii) creating enzyme aggregates through protein‐scaffold‐based self‐assembly or synthetic chemistry, etc.^[^ ^2^ ^]^ However, protein engineering through direct evolution or rational design requires a significant workload or a profound understanding of catalytic mechanisms.^[^ ^3^ ^]^ Furthermore, due to the well‐known stability‐activity trade‐off, particularly when the mutation site resides close to the active site, achieving equilibrium between enzyme activity and stability poses an exceedingly formidable task.^[^ ^4^ ^]^ To address these challenges, functional protein components have been developed, achieving different biological purposes by fusing different structural domains. For instance, the orthogonal Spytag/Spycatcher system facilitates the assembly of different components through hetero‐peptide bond formation.^[^ ^5^ ^]^ Nevertheless, such assemblies are greatly influenced by micro‐environmental factors like temperature and pH, which can significantly affect assembly efficiency.^[^ ^6^ ^]^ Therefore, the development of a simple yet multifunctional protein module is crucial.

Self‐assembling proteins represent a unique class with inherent structural and functional capabilities.^[^ ^7^ ^]^ They can spontaneously adopt specific configurations or organize them into higher‐order structures. For example, amyloid nanofibrils represent straight, unbranched peptide/protein fibrous structures, originating from a nucleation process. This process commences with the self‐assembly of soluble amyloidogenic peptides or proteins into protofibrils, onto which additional amyloidogenic peptides or proteins aggregate, forming highly ordered β sheet structures, ultimately leading to the formation of mature amyloid fibers.^[^ ^8^ ^]^ While these protein scaffolds have shown promise in facilitating enzyme assembly, challenges remain regarding their direct fusion with guest proteins. In many instances, the direct genetic fusion of scaffolds with guest proteins or enzymes may impede the assembly process in vivo, as large guest proteins or enzymes can introduce spatial hindrances to the system. To overcome this issue, the genetic fusion variant should be co‐fibrillated in vitro with a carrier protein, which is the wild‐type scaffold protein alone.^[^ ^9^ ^]^ Moreover, direct fusion of guest proteins or enzymes with self‐assembling protein scaffolds often leads to a reduction in catalytic efficiency.^[^ ^10^ ^]^ To address this, an alternative method is to forego direct fusion and instead use click chemistry or bio‐orthogonal systems, such as the SpyTag/SpyCatcher system, to link the scaffold protein and the guest protein in vitro. However, this method reduces the loading capacity of the guest protein and increases the cost and complexity of the process.^[^ ^11^ ^]^ Therefore, it highlights the necessity of developing a versatile, easy‐to‐use self‐assembling protein scaffold that enables direct in vivo assembly of guest proteins without chemical or bio‐conjugation, thus advancing biotechnological applications.

The gamma‐prefoldin (γ‐PFD) protein, derived from *Methanococcus jannaschii*, functions as a molecular chaperone and features fibrous structures formed extensively through β‐sheets.^[^ ^12^ ^]^ The monomer of γ‐PFD dimerizes, and these dimers stack to create a multilayered fibrous structure in vivo.^[^ ^13^ ^]^ Although γ‐PFD has been used as a scaffold for enzyme immobilization, direct genetic fusion of guest proteins was found to be unfavorable due to potential disruptions in filament assembly. To overcome this, in vitro protein‐peptide bio‐conjugation techniques are employed for target enzyme immobilization, which raises concerns about low loading capacity and conjugation efficiency.^[^ ^14^ ^]^

In this study, we engineered a thermophilic extension‐resistant mutant (TERM) of γ‐PFD,^[^ ^15^ ^]^ featuring seven consecutive alanine mutations in its β‐sheet regions which originally intended to inhibit γ‐PFD filament elongation, into an effective self‐assembling scaffold for direct in vivo guest protein assembly without the need for chemical or bio‐conjugation modifications in vitro. To enhance the structural integrity of TERM as a self‐assembly scaffold, disulfide bonds were introduced to create a stable TERM‐M67C mutant. Subsequently, the monomeric enzyme Endo‐1,4‐beta‐xylanase A from *Aspergillus niger* (XynA) was directly fused with TERM‐M67C, resulting in intact, rod‐shaped polymeric proteins with improved activity and stability.^[^ ^16^ ^]^ To further demonstrate the ability of TERM‐M67C to enhance the catalytic performance of guest proteins and accommodate complex multimeric proteins, the hetero‐tetrameric metalloenzyme nitrile hydratase from *Pseudomonas putida* (*Pp*NHase) was fused to TERM‐M67C.^[^ ^17^ ^]^ This chimeric protein assembled into oligomers and significantly overcame the typical stability‐activity trade‐off of such industrially relevant enzymes. Molecular dynamics (MD) simulations showed that the TERM‐M67C scaffold provides substantial protection to the core assembly by polymerizing the guest protein. Additionally, when fluorescent proteins mVenus and mCerulean3 were attached to the N‐terminus and C‐terminus of the TERM‐M67C scaffold, strong Förster resonance energy transfer (FRET) was detected, highlighting TERM‐M67C's potential as a highly effective enzyme cascade platform.^[^ ^18^ ^]^

## 2 Results and Discussion

### 2.1 Disulfide Bond Introduction to Enhance Self‐Assembly Capability and Stability of the TERM Scaffold

**Figure 1:**
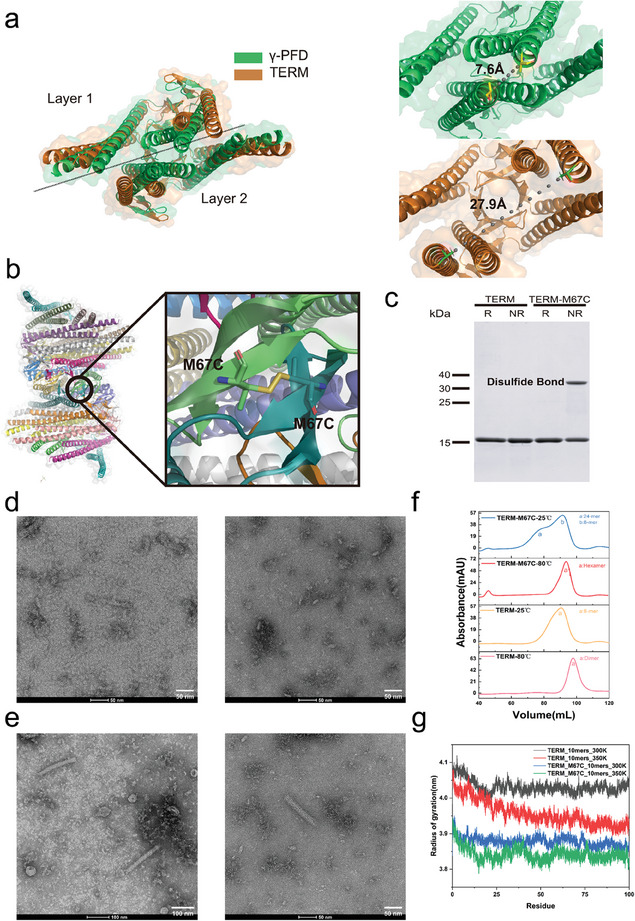
The introduction of disulfide bonds enhances the assembly regularity and stability of TERM. a) The structural alignment of γ‐PFD and TERM. The structure of γ‐PFD was downloaded from the RCSB Protein Data Bank (PDB ID: 6vy1). The modeled structure of TERM was constructed using AlphaFold3 server. The distance between their respective C‐termini was measured using the measurement wizard from the PyMOL menu. b) The introduction of the M67C mutation into the TERM monomer forms inter‐subunit disulfide bonds. c) The SDS‐PAGE analysis of the WT TERM and TERM‐M67C (0.5 mg mL^−1^) under reducing (*R*) and non‐reducing (NR) conditions. The molecular weight of the TERM monomer is 17 kDa, while TERM‐M67C forms a 34 kDa dimer under non‐reducing conditions due to the presence of disulfide bonds. 2‐Mercaptoethanol was used as the reducing agent. d) Transmission electron microscopy (TEM) images of the WT TERM. e) TEM images of the TERM‐M67C. f) Size exclusion chromatography (SEC) determination of the WT TERM and TERM‐M67C at room temperature (25 °C) and after heat treatment at 80 °C for 20 min. g) Radius of gyration of the WT TERM and TERM‐M67C under 300 and 350 K during 100 ns simulation.

TERM underwent a series of alanine mutations in the β‐sheet region, which weakened the interactions between the β‐sheet segments, thereby preventing it from stacking into a multilayered fibrous structure like γ‐PFD (Figure S1, Supporting Information).^[^ ^12^ ^]^ To compare the structural differences between TERM and γ‐PFD, the multimeric structure of TERM was generated using the AlphaFold3 server^[^ ^19^ ^]^ and aligned with the CryoEM structure of γ‐PFD (PDB ID: 6vy1).^[^ ^20^ ^]^ Unlike γ‐PFD, TERM displayed a less compact assembly. The distance between the C‐termini of TERM monomers in adjacent layers (25.6 Å) is significantly greater than that of γ‐PFD (7.6 Å) (**Figure** **1**a). Additionally, MD simulations reveal that TERM (tetramer) exhibits a larger radius of gyration (Rg) at 300 K than γ‐PFD (Figure S2, Supporting Information). These findings suggest that TERM adopts a looser structure, characterized by reduced assembly precision and less stable configurations. Despite this, the less compact structure of TERM may offer opportunities for modifications that enable it to directly accommodate target guest proteins, as the looser structure at the C‐termini could provide space for better accommodation of bulky guest proteins between different layers.

Here, we aim to enhance the assembly and stability of the TERM protein by introducing inter‐chain interactions between TERM monomers, while preserving the loose C‐terminal structure to accommodate target guest proteins. To achieve this, Disulfide by Design 2 was used to predict potential sites for disulfide bond formation between TERM monomers (Table S1, Supporting Information).^[^ ^21^ ^]^ We constructed the three mutants, finding that the mutant TERM‐M67C successfully facilitated disulfide bond formation as confirmed by non‐reduced/reduced SDS‐PAGE analysis (Figure 1b,c). The extent of disulfide bond formation was then quantified using the Ellman method,^[^ ^22^ ^]^ showing that ≈0.39 moles of disulfide bonds form per mole of TERM‐M67C variant (Figure S3; Table S2, Supporting Information). Transmission Electron Microscopy (TEM) analysis indicated that, although TERM‐M67C fibers were not as long as γ‐PFD fibers, they exhibited a higher degree of assembly compared to the unmodified TERM protein.^[^ ^15^ ^]^ The introduction of disulfide bonds resulted in a more ordered and compact assembly of the TERM‐M67C scaffold, characterized by straight rod‐like structures ranging from 100 to 200 nm in length, in contrast to the irregular aggregates of the wild‐type TERM protein (Figure 1d).

To further investigate the impact of disulfide bonds on the stability of the TERM‐M67C scaffold, the mutant was heat‐treated at 80 °C for 20 min. Changes in the assembly state of TERM‐M67C post‐treatment were analyzed using size‐exclusion chromatography (SEC) and compared to that of the WT TERM. SEC results indicated that prior to heat treatment, the TERM scaffold primarily existed as 8‐mer, but after exposure to 80 °C for 20 min, its assembly completely disintegrated to its dimeric form. In contrast, the mutant TERM‐M67C not only showed a higher degree of assembly than TERM (ranging from 8‐ to 24‐mer) but also retained its hexameric assembly after the heat treatment. These SEC results demonstrated that the introduction of disulfide bonds enhances the structural stability of the protein scaffold assembly (Figure 1e; Figure S4, Supporting Information).

### 2.2 Structural Insights Unraveling the Stability Mechanism of TERM‐M67C

To gain insights into the stability difference between TERM‐M67C and its parent protein, MD simulations were performed on the tetramer structures of the WT TERM and TERM‐M67C. After 100 ns of simulation, the RMSF analysis reveals that the presence of disulfide bonds diminishes the fluctuation observed in the β‐sheet region of TERM‐M67C (Figure S5, Supporting Information). Furthermore, the WT TERM undergoes helical unfolding at 350 K (Figure S6, Supporting Information). In addition, the Radius of gyration (Rg) of the mutant TERM‐M67C was lower than that of WT TERM at both 300 and 350 K (Figure 1g). The lower *R*g suggests that, even under high‐temperature conditions, TERM‐M67C is better able to maintain its three‐dimensional structure, potentially explaining its increased assembly and stability.

Further analysis of hydrogen bonds revealed that the introduction of disulfide bonds significantly increased the number of internal hydrogen bonds within the tetramer of TERM‐M67C(10‐mer). At 350 K, TERM‐M67C generated an average of 433 hydrogen bonds per frame, compared to 395 in WT TERM (Figure S7, Supporting Information). The increased number of hydrogen bonds is also a contributing factor to the enhanced stability of TERM‐M67C. Subsequently, we calculated the binding free energy between the two adjacent layers of the WT TERM and the mutant TERM‐M67C under 350 K. In the TERM tetramer, the binding free energy between the two adjacent layers was −92.17 kcal mol^−1^, whereas in the TERM‐M67C tetramer, it was −97.54 kcal mol^−1^ (Figure S8, Supporting Information). The lower binding free energy of the two dimers in the TERM‐M67C tetramer indicates a tighter interaction between different TERM‐M67C layers.

### 2.3 Enhancing the Catalytic Performance of Monomeric Enzyme XynA via TERM‐M67C Scaffold

After successfully enhancing the stability and self‐assembly capabilities of the TERM protein by introducing inter‐chain disulfide bonds, we proceeded to fuse suitable guest enzymes with the TERM‐M67C mutant to investigate whether such fusion could improve the catalytic performance of target guest enzymes through scaffold‐guided self‐assembly. In many industrial catalytic processes, the enhancement of enzyme thermal stability could help enzymes withstand higher catalytic temperatures, thereby accelerating the catalytic reaction process. By utilizing the innate high thermostability and self‐assembly ability of TERM‐M67C, we aim to develop a durable enzyme scaffold that can overcome the stability‐activity trade‐off exhibited by industrial enzymes.

The monomeric enzyme XynA from *A. niger* was selected as the guest protein. XynA catalyzes the hydrolysis of internal β−1,4‐glycosidic bonds within xylan under acidic conditions, producing xylo‐oligosaccharides like xylobiose and xylotetraose. XynA holds significant promise for various industrial applications, including biomass degradation, papermaking, and food processing.^[^ ^23^ ^]^ However, its relatively low thermal stability and catalytic activity have limited its broader industrial use.

**Figure 2:**
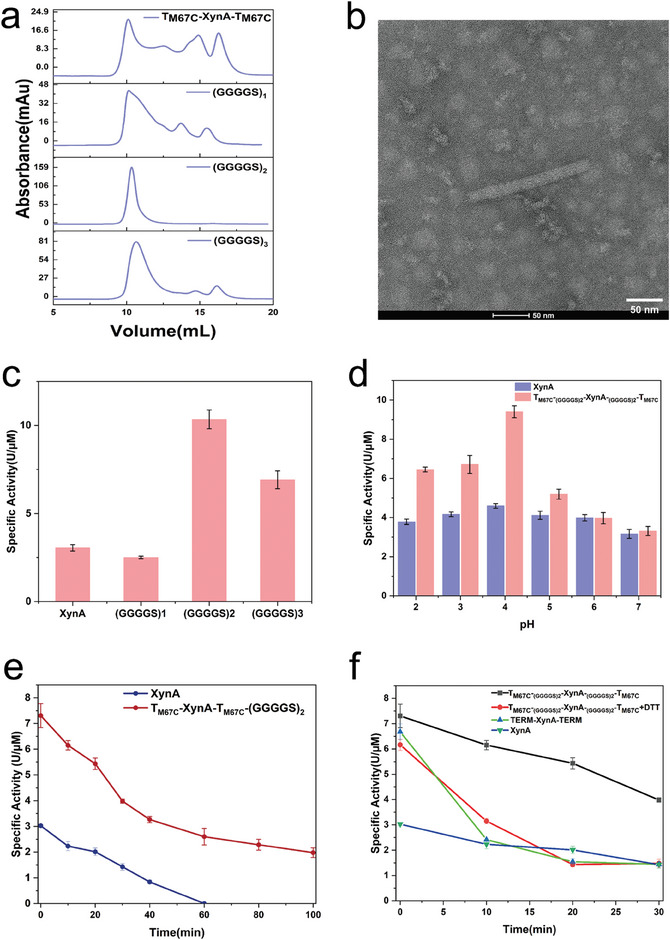
The monomeric enzyme XynA forms highly polymerized structures via the TERM‐M67C guided self‐assembly with better catalytic performance. a) Size Exclusion Chromatography (SEC) analysis of four chimeric proteins: T_M67C_‐XynA‐T_M67C_, T_M67C_‐_(GGGGS)1_‐XynA‐_(GGGGS)1_‐T_M67C_ (‐(GGGGS)_1_), T_M67C_‐_(GGGGS)2_‐XynA‐_(GGGGS)2_‐T_M67C_ (‐(GGGGS)_2_) and T_M67C_‐_(GGGGS)3_‐XynA‐_(GGGGS)3_‐T_M67C_ (‐(GGGGS)_3_). b) Transmission electron microscopy (TEM) images of T_M67C_‐_(GGGGS)2_‐XynA‐_(GGGGS)2_‐T_M67C_. The scale bar is 50 nm. The sample was negative‐stained using uranyl acetate. c) Specific enzymatic activities of the WT XynA and T_M67C_‐_(GGGGS)2_‐XynA‐_(GGGGS)2_‐T_M67C_ using Xylan as substrate. d) The comparison of specific activities of the WT XynA and T_M67C_‐_(GGGGS)2_‐XynA‐_(GGGGS)2_‐T_M67C_ under different acidic pH conditions. e) The residual activities of the WT XynA and T_M67C_‐_(GGGGS)2_‐XynA‐_(GGGGS)2_‐T_M67C_ after heat treatment under 55 °C. f) The residual activities of the WT XynA and T_M67C_‐_(GGGGS)2_‐XynA‐_(GGGGS)2_‐T_M67C_ with and without DTT treatment. The samples were treated under 55 °C for 30 min.

By fusing the N‐terminus and C‐terminus of XynA with individual TERM‐M67C monomers, we obtained the chimeric enzyme TERM‐M67C‐XynA‐TERM‐M67C (T_M67C_‐XynA‐T_M67C_), with an approximate molecular weight of 54 kDa (Figure S9, Supporting Information). SEC was then applied to examine the polymerization profile of T_M67C_‐XynA‐T_M67C_. The results indicated that while direct fusion succeeded in forming some high molecular weight aggregates (>600 kDa), a sizable portion of the protein remained in lower oligomeric states or even as monomers (**Figure** **2**a). We hypothesize that the lower degree of aggregation is due to steric hindrance caused by the direct fusion of XynA, which impedes further polymerization of T_M67C_‐XynA‐T_M67C_.

To address this issue, flexible linkers (GGGGS)_n_ were introduced to optimize the fusion between XynA and TERM‐M67C. Flexible linkers can provide the guest protein with additional spatial freedom, reducing steric hindrance.^[^ ^24^ ^]^ We constructed fusion proteins with three different lengths of linkers, resulting in three different chimeric enzymes: T_M67C_‐_(GGGGS)1_‐XynA‐_(GGGGS)1_‐T_M67C_, T_M67C_‐_(GGGGS)2_‐XynA‐_(GGGGS)2_‐T_M67C_, and T_M67C_‐_(GGGGS)3_‐XynA‐_(GGGGS)3_‐T_M67C_ (Figure S10, Supporting Information). SEC analysis revealed that the (GGGGS)_2_ linker was the most effective in facilitating the assembly of XynA into high molecular weight polymers exceeding 600 kDa (Figure 2a; Figure S11, Supporting Information). This suggests that a medium‐length flexible linker may effectively reduce steric hindrance, thereby promoting efficient assembly of the target protein. TEM analysis showed that the purified T_M67C_‐_(GGGGS)2_‐XynA‐_(GGGGS)2_‐T_M67C_ still assembled into short rod‐like structures (Figure 2b).

To further explore how scaffold‐guided guest protein assembly affects functionality, the catalytic activities of the WT XynA, T_M67C_‐_(GGGGS)1_‐XynA‐_(GGGGS)1_‐T_M67C_, T_M67C_‐_(GGGGS)2_‐XynA‐_(GGGGS)2_‐T_M67C_ and T_M67C_‐_(GGGGS)3_‐XynA‐_(GGGGS)3_‐T_M67C_ were measured. The results showed that T_M67C_‐_(GGGGS)2_‐XynA‐_(GGGGS)2_‐T_M67C,_ which forms uniform high molecular weight polymers (Figure 2a), exhibited the highest catalytic activity, about four times that of the WT enzyme (Figure 2c). The kinetic parameters of the WT XynA and T_M67C_‐_(GGGGS)2_‐XynA‐_(GGGGS)2_‐T_M67C_ were then determined. The *K*_m_ value of T_M67C_‐_(GGGGS)2_‐XynA‐_(GGGGS)2_‐T_M67C_ was 21.58 mm, lower than that of its parent enzyme (24.74 mM). The *k*_cat_ value of T_M67C_‐_(GGGGS)2_‐XynA‐_(GGGGS)2_‐T_M67C_ increased significantly to 12.69 s⁻¹, approximately double that of its parent enzyme (**Table** **1** and Figure S12, Supporting Information).

**Table 1:** The kinetic parameters of XynA and T_M67C_‐_(GGGGS)2_‐XynA‐_(GGGGS)2_‐T_M67C_.

Enzyme	Specific activity [U mm ^−1^]	*K*_m_ [mm]	*k*_cat_ [s^−1^]	*k*_cat_ */K*_m_ [s^−1^mM^−1^]
XynA	4.59	24.74	5.85	0.24
(GGGGS)_2_ ^a)^	10.33	21.58	12.69	0.59

^a)^ the fusion protein:T_M67C_‐_(GGGGS)2_‐XynA‐_(GGGGS)2_‐T_M67C_.

XynA is an enzyme that catalyzes reactions under acidic conditions, with an optimal pH of 4.^[^ ^25^ ^]^ Further analysis under various pH conditions showed that T_M67C_‐_(GGGGS)2_‐XynA‐_(GGGGS)2_‐T_M67C_ had markedly enhanced catalytic activity in acidic conditions (pH 2‐5) compared to the WT enzyme (Figure 2d). This suggests that the highly assembled state of T_M67C_‐_(GGGGS)2_‐XynA‐_(GGGGS)2_‐T_M67C_ may stabilize its structure in low pH environments, improving catalytic efficiency.

Additionally, we evaluated the stability of T_M67C_‐_(GGGGS)2_‐XynA‐_(GGGGS)2_‐T_M67C_ under a thermal environment. After incubating the WT enzyme and T_M67C_‐_(GGGGS)2_‐XynA‐_(GGGGS)2_‐T_M67C_ at 55 °C for 60 min, the WT enzyme lost catalytic activity completely, while T_M67C_‐_(GGGGS)2_‐XynA‐_(GGGGS)2_‐T_M67C_ retained 36% of its initial activity, with specific activity comparable to the non‐heat‐treated WT enzyme (Figure 2e).

To investigate whether the introduction of inter‐chain disulfide bonds helps in improving the thermostability of the chimeric enzyme, TERM‐XynA‐TERM, T_M67C_‐_(GGGGS)2_‐XynA‐_(GGGGS)2_‐T_M67C_ treated with dithiothreitol (DTT), and T_M67C_‐_(GGGGS)2_‐XynA‐_(GGGGS)2_‐T_M67C_ without DTT treatment were subjected to heat treatment under 55 °C (Figure 2f). Both the DTT‐reduced T_M67C_‐_(GGGGS)2_‐XynA‐_(GGGGS)2_‐T_M67C_ and TERM‐XynA‐TERM, despite improved activity relative to the WT enzyme, showed similarly poor stability as the parent enzyme after heat treatment, underscoring the crucial role of disulfide bonds in maintaining thermal stability.

### 2.4 Mechanistic Insights into the Enhanced Catalysis Activity and Stability of T_M67C_‐_(GGGGS)2_‐XynA‐_(GGGGS)2_‐T_M67C_

**Figure 3:**
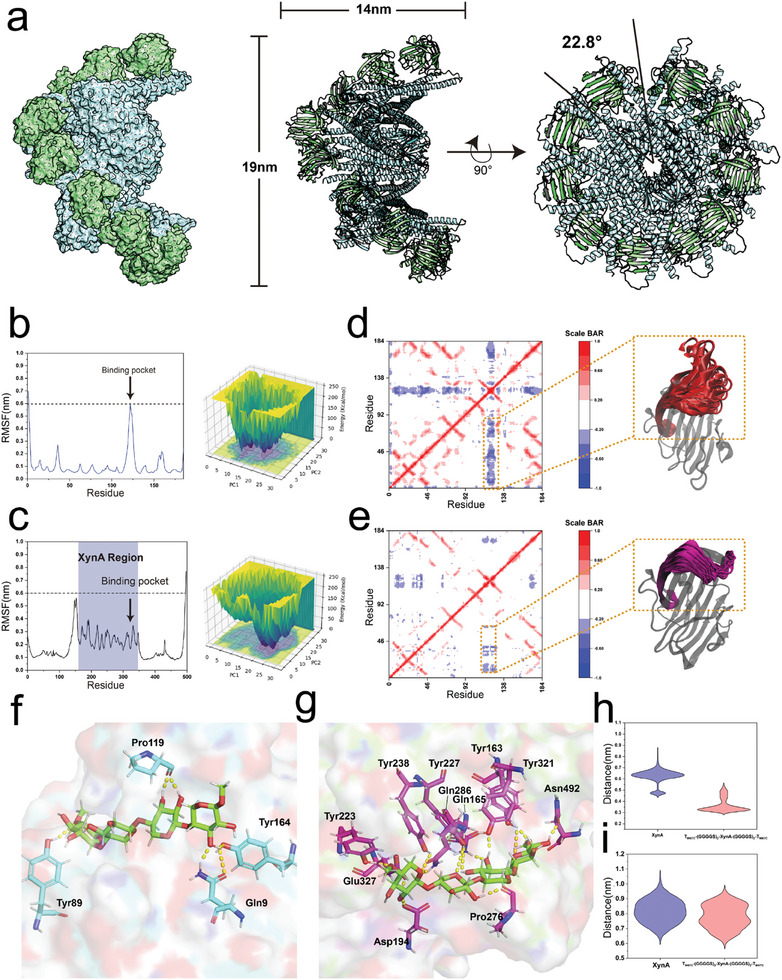
Mechanistic insights into the enhanced stability and activity of T_M67C_‐(GGGGS)_2_‐XynA‐(GGGGS)_2_‐T_M67C_ through static and dynamic analyses. a) The modeled structure of T_M67C_‐_(GGGGS)2_‐XynA‐_(GGGGS)2_‐T_M67C_ predicted by AlphaFold3 server. The TERM‐M67C scaffold was shown in Palecyan. The XynA was shown in pale green. Within this assembly, each protein forms an angle of 28° with the centroid. b) RMSF and Free Energy Landscape of the WT XynA under 350 K. The black arrow points to the region of the substrate binding pocket of the WT XynA. c) RMSF and Free Energy Landscape of the XynA immobilized on TERM‐M67C under 350 K. The black arrow points to the region of the substrate binding pocket of the XynA immobilized on TERM‐M67C. The indigo‐filled part represents the XynA region in T_M67C_‐_(GGGGS)2_‐XynA‐_(GGGGS)2_‐T_M67C_. d) Dynamic Cross‐Correlation Matrix (DCCM) of the WT XynA at 350 K. The substrate binding region showing significant negative correlations was highlighted in the red cartoon. e) DCCM of the XynA immobilized on TERM‐M67C under 350 K. The substrate binding region showing reduced correlations was highlighted in the purple cartoon. f) The docking of Xylan into the substrate binding pocket of the WT XynA. The substrate was shown in green sticks. The residues participating in substrate binding were presented as cyan sticks. g) The docking of Xylan into the substrate binding pocket of T_M67C_‐_(GGGGS)2_‐XynA‐_(GGGGS)2_‐T_M67C_. The substrate was shown in green sticks. The residues participating in substrate binding were presented as purple sticks. The hydrogen bonds were shown as yellow dashed lines. h) The distance distribution between the side‐chain oxygen atom of Glu236 and Glu79 to the carbon atom of the catalytic group in the WT XynA and T_M67C_‐_(GGGGS)2_‐XynA‐_(GGGGS)2_‐T_M67C_. i) The distance distribution between the side‐chain O atom of Glu236/Glu79 (catalytic site) and the H atom of Glu327/Glu170 (proton donor) of the WT XynA and T_M67C_‐_(GGGGS)2_‐XynA‐_(GGGGS)2_‐T_M67C_.

To further explore the mechanism behind the improved catalytic performance of T_M67C_‐_(GGGGS)2_‐XynA‐_(GGGGS)2_‐T_M67C_, its 3D structure was constructed using the AlphaFold3 server. According to the model, T_M67C_‐_(GGGGS)2_‐XynA‐_(GGGGS)2_‐T_M67C_ is a helical twist with XynA monomers accommodated uniformly on the outer surface of the filament. The outer diameter of the filament is ≈14 nm (**Figure** **3**a). MD simulations were performed for both the WT XynA and T_M67C_‐_(GGGGS)2_‐XynA‐_(GGGGS)2_‐T_M67C_ at 350 K. RMSF analysis of the simulation trajectories revealed that the top and bottom layers of the T_M67C_‐_(GGGGS)2_‐XynA‐_(GGGGS)2_‐T_M67C_ fibril, exposed to the solvent, showed significant fluctuations at 350 K. In contrast, the dimer layers closer to the filament core exhibited greater stability (Figure S13, Supporting Information). Our analysis then focused on the central filament layers as representative samples. RMSF analysis of the WT XynA highlighted notable fluctuations in residues 115–135, which encompass the substrate binding region (Figure 3b). Such fluctuations could potentially impact the stability of substrate binding. Conversely, T_M67C_‐_(GGGGS)2_‐XynA‐_(GGGGS)2_‐T_M67C_ displayed remarkable structural stability in this region, with RMSF values below 0.3 nm, compared to 0.6 nm in the WT, indicating greater stability in the engineered protein's substrate binding region (Figure 3c).

To verify this observation, we compared the free energy landscapes of WT XynA and T_M67C_‐_(GGGGS)2_‐XynA‐_(GGGGS)2_‐T_M67C_. The results demonstrated that T_M67C_‐_(GGGGS)2_‐XynA‐_(GGGGS)2_‐T_M67C_ exhibited a single free energy well at 350 K, in stark contrast to WT XynA's energy distribution (Figure 3b,c). The presence of a single, distinct lowest free energy well generally signifies that the system has one stable state or conformation. This single well indicates that the system preferentially occupies this state, as it represents the point of lowest free energy, suggesting that it is the most thermodynamically stable state.^[^ ^26^ ^]^ This suggests that the engineered protein maintains a more stable structure under 350 K and is more likely to remain in the lowest energy state, providing thermodynamic evidence for its enhanced stability.

Additionally, dynamic cross‐correlation matrix (DCCM) analysis was conducted to assess cooperative movements within structural regions of both proteins. The WT XynA showed significant negative correlations in the substrate binding region, pointing to extensive cooperative fluctuations (Figure 3d). On the other hand, T_M67C_‐_(GGGGS)2_‐XynA‐_(GGGGS)2_‐T_M67C_ displayed reduced correlations in the same region, indicating more coordinated residue movements consistent with its structural stability (Figure 3e).^[^ ^27^ ^]^

Further, we docked the substrate into the substrate binding pockets of both WT XynA and T_M67C_‐_(GGGGS)2_‐XynA‐_(GGGGS)2_‐T_M67C_. It was reported that under acidic conditions, the catalytic residues of WT XynA, Glu79, and Glu170 (corresponding to Glu236 and Glu327 in T_M67C_‐_(GGGGS)2_‐XynA‐_(GGGGS)2_‐T_M67C_), are protonated and deprotonated, respectively. Glu79 acts in nucleophilic attack, while Glu170 acts as a proton donor.^[^ ^28^ ^]^ In WT XynA, docking revealed hydrogen bonds between Glu79, Tyr89, Pro119, and Tyr164 (Figure 3f; Figure S14, Supporting Information). However, in the case of T_M67C_‐_(GGGGS)2_‐XynA‐_(GGGGS)2_‐T_M67C_, additional hydrogen bonds were formed between the substrate and protein, among them, Pro276 and Gln286 are located in the substrate‐binding region. (Figure 3g). The presence of more hydrogen bonds indicates a stronger substrate binding in the engineered protein. Notably, Asn492, an amino acid from the TERM‐M67C protein scaffold, also participated in substrate binding, which might aid in orienting the substrate in the active site.

Following this, MD simulations were performed for both the WT XynA and T_M67C_‐_(GGGGS)2_‐XynA‐_(GGGGS)2_‐T_M67C_ with their substrate. Using the MM/PBSA method,^[^ ^29^ ^]^ we determined the receptor‐ligand binding free energies, which were −32.56 kcal mol^−1^ for WT XynA and −49.56 kcal mol^−1^ for T_M67C_‐_(GGGGS)2_‐XynA‐_(GGGGS)2_‐T_M67C_ (Figure S15, Supporting Information), correlating with measured *K*_m_ value. The distance between the side‐chain oxygen atoms of the catalytic residue (Glu79 in the WT XynA and the corresponding Glu236 in T_M67C_‐_(GGGGS)2_‐XynA‐_(GGGGS)2_‐T_M67C_) and the carbon atoms on the hydrolyzed β−1,4‐glycosidic bond was then calculated. The results showed that the side‐chain oxygen atom of Glu236 in T_M67C_‐_(GGGGS)2_‐XynA‐_(GGGGS)2_‐T_M67C_ was closer to the carbon atom of the catalytic group, with an average distance of 0.32 nm, compared to 0.64 nm in WT XynA (Figure 3h). Similarly, proton donor Glu327's hydrogen atoms in T_M67C_‐_(GGGGS)2_‐XynA‐_(GGGGS)2_‐T_M67C_ were closer to Glu236's oxygen atoms at 0.77 and 0.83 nm in WT XynA (Figure 3i).

### 2.5 TERM‐M67C Scaffold Guides Multimeric NHase to Form Higher‐Order Structure With Improved Catalytic Performance

The successful fusion of the monomeric enzyme XynA demonstrated that TERM‐M67C, as a scaffold protein, not only facilitates the polymerization of guest proteins without compromising its own assembly properties but also enhances their catalytic performance. However, most enzymes in nature exist in multimeric forms. Consequently, another well‐industrialized NHase from *P. putida* (*Pp*NHase) was selected as a guest enzyme. NHase usually exists as a hetero‐tetramer αββα consisting of two α subunits and two β subunits and is crucial in the production of bulk chemicals such as acrylamide.^[^ ^30^ ^]^

**Figure 4:**
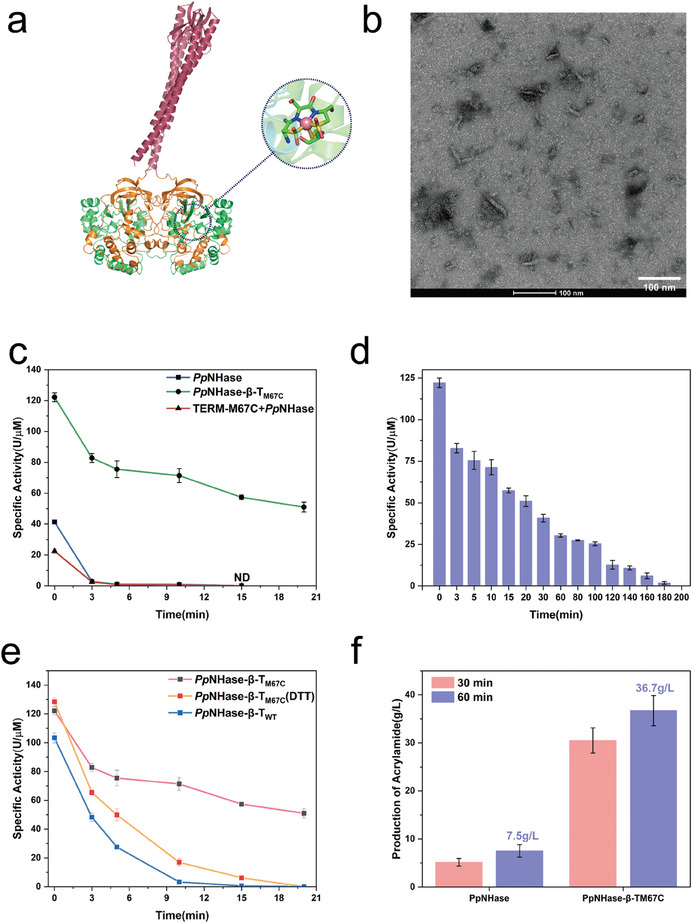
Fusion of TERM‐M67C with the multimeric protein NHase enhances its catalytic performance. a) Schematic representation of the structure of the fusion protein *Pp*NHase‐β‐T_M67C_. The orange part represents the β subunit of NHase, and the green part represents the α subunit. The active center of NHase is located between the α and β subunits. TERM‐M67C was fused to the C‐terminus of the β subunit, forming a disulfide bond between the TERM‐M67C dimers. b) Typical configuration of *Pp*NHase‐β‐T_M67C_ observed under TEM, showing a short rod‐like structure. c) Changes in specific enzyme activity after heat treatment at 65 °C for *Pp*NHase, *Pp*NHase‐β‐T_M67C_, and the mixture of TERM‐M67C and *Pp*NHase. d) Inactivation test of *Pp*NHase‐β‐T_M67C_. The enzyme was heat‐treated at 65 °C, showing complete inactivation after 200 min. e) Thermal stability assessment of enzymes at 65 °C. Three samples were compared: *Pp*NHase‐β‐T_WT_ without disulfide bonds, *Pp*NHase‐β‐T_M67C_ treated with DTT to reduce disulfide bonds, and untreated *Pp*NHase‐β‐T_M67C_. f) Whole‐cell catalytic conversion of acrylonitrile by *Pp*NHase and *Pp*NHase‐β‐T_M67C_.

The TERM‐M67C was firstly fused to either the N‐terminus or C‐terminus of the α subunit or β subunit of *Pp*NHase linked by (GGGGS)_2_ linker, resulting in four variants: *Pp*NHase‐T_M67C_‐α, *Pp*NHase‐α‐T_M67C_, *Pp*NHase‐T_M67C_‐β, *Pp*NHase‐β‐T_M67C_. The activity of these variants was evaluated, revealing that *Pp*NHase‐β‐T_M67C_ achieved an activity of 122.15 U µm ^−1^ (**Table** **2**), three times higher than the WT *Pp*NHase (41.37 U/ µm ^−1^), whereas the activities of the variants *Pp*NHase‐T_M67C_‐α, *Pp*NHase‐α‐T_M67C_, *Pp*NHase‐T_M67C_‐β were significantly reduced (Figures S16 and S17, Supporting Information). NHase maturation involves a self‐subunit swapping mechanism where the α subunit from the apo‐NHase exchanges with that of the holo‐αe_2_ complex (Figure S18, Supporting Information).^[^ ^31^ ^]^ The direct fusion of TERM‐M67C with the α subunit might interrupt the self‐subunit swapping process and thus cause a decrease in activity. In addition, since the N‐terminus of the β subunit was buried within the tetrameric interface and engaged in inter‐subunit interactions, fusion with TERM‐M67C might also have a negative effect on the catalytic activity. Therefore, we selected the C‐terminus of the β subunit as the TERM‐M67C fusion site (**Figure** **4**a).

**Table 2:** The kinetic parameters of *Pp*NHase and *Pp*NHase‐β‐T_M67C_.

Enzyme	Specific activity [U mm ^−1^]	*K*_m_ [mm]	*k*_cat_ [s^−1^]	*k*_cat_ */K*_m_ [s^−1^mM^−1^]
*Pp*NHase	41.37	118.8	123.8	1.04
*Pp*NHase‐β‐T_M67C_	112.15	61.9	398.6	6.44

To elucidate the reasons for the enhancement in activity of *Pp*NHase‐β‐T_M67C_, the kinetic parameters of the WT *Pp*NHase and *Pp*NHase‐β‐T_M67C_ were evaluated. The *K*_m_ value of *Pp*NHase‐β‐T_M67C_ (64.1 mm) decreased to almost half of the WT *Pp*NHase (118.8 mm), while the *k*_cat_ value of *Pp*NHase‐β‐T_M67C_ (398.6 s^−1^) is three times that of its parent enzyme (123.8 s^−1^) (Table 2; Figure S19, Supporting Information). The results indicate that the fusion of TERM‐M67C and *Pp*NHase not only enhances the affinity between the enzyme and substrate but also improves the catalytic efficiency of the enzyme. SEC analysis was subsequently conducted, revealing that *Pp*NHase‐β‐T_M67C_ exhibits a higher molecular weight (exceeding 600 kDa) compared to the WT *Pp*NHase, which is known to function as a hetero‐tetramer with a molecular weight of ≈100 kDa^[^ ^32^ ^]^ (Figure S20, Supporting Information). SDS‐PAGE confirmed the correct formation of disulfide bonds within TERM‐M67C after fusion with *Pp*NHase. TEM revealed that *Pp*NHase‐β‐T_M67C_ forms a short rod‐like structure ≈100 nm long and 20 nm wide, indicating the formation of a higher‐order structure (Figure 4b).

Thermal stability assessments conducted at 65 °C demonstrated the exceptional thermal stability of *Pp*NHase‐β‐T_M67C_. At this temperature, the half‐life of the WT *Pp*NHase is 0.49 min, whereas *Pp*NHase‐β‐T_M67C_ significantly prolongs to 14.05 min, surpassing *Pp*NHase by 28‐fold (Figure 4c; Figure S21, Supporting Information). Additionally, while *Pp*NHase loses its activity entirely after heat treatment for 10 min, *Pp*NHase‐β‐T_M67C_ maintains activity for 180 min (Figure 4d).

To understand the stability enhancement, *Pp*NHase, *Pp*NHase‐β‐T_wt_, and *Pp*NHase‐β‐T_M67C_ were treated with 10 mm DTT. After DTT treatment, the stability of either *Pp*NHase‐β‐T_WT_, lacking disulfide bonds, or DTT‐treated *Pp*NHase‐β‐T_M67C_ was comparable to the WT *Pp*NHase (Figure 4e). Therefore, the enhanced activity and stability of *Pp*NHase‐β‐T_M67C_ may be attributed to the self‐assembling structure of TERM‐M67C formed through disulfide bond introduction. This catalytic performance improvement aligns with the increased activity and stability observed in the fusion of TERM‐M67C with XynA.

In the industrial production of acrylamide, production efficiency is one of the most critical factors.^[^ ^33^ ^]^ Enzymes in industrial environments not only require high activity but also demand exceptional stability. We assessed acrylamide production efficiency by simulating the industrial process. Initially, 23 mL of acrylonitrile was added to a reactor, and then 27 mL of cell resuspension harboring *Pp*NHase‐β‐T_M67C_ or *Pp*NHase was added to achieve a final concentration of OD_600_ = 5. After a 1 h reaction, the final yield of acrylamide by *Pp*NHase‐β‐T_M67C_ reached 36.7 g L^−1^, a fivefold increase compared to *Pp*NHase (Figure 4f). This underscores the substantial potential of the TERM‐M67C scaffold in practical applications.

### 2.6 Mechanism Analysis of the Enhanced Catalytic Performance of *Pp*NHase‐β‐T_M67C_

**Figure 5:**
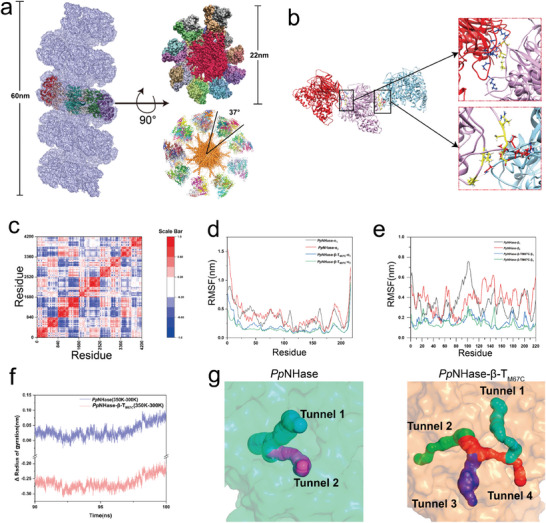
Investigation of the mechanism of the enhanced catalysis performance of *Pp*NHase‐β‐T_M67C_. a) The helical twist structure of *Pp*NHase‐β‐T_M67C_ (37‐mer). The structure has a total length of 60 nm, with each layer (10‐mer) a diameter of 22 nm. Within each layer, the monomers of *Pp*NHase‐β‐T_M67C_ have an inter‐monomer angle of ≈37°. The structure was constructed using AlphaFold3 server. b) The new protein‐protein interactions that formed between the tetrameric interfaces of *Pp*NHase‐β‐T_M67C_ tetrameric units during simulations at the lowest free energy conformations. Three *Pp*NHase tetramers extracted from the helical twist structure of *Pp*NHase‐β‐T_M67C_ were shown in red, light pink, and pale cyan cartoons, respectively. The interface amino acid residues were shown as sticks. c) The DCCM matrix calculated from the trajectories of five *Pp*NHase tetrameric units extracted from molecular dynamics simulations of the pentameric model *Pp*NHase‐β‐T_M67C_. This represents the correlation in movement among the five NHase molecules. d) The RMSF of the α‐subunit of *Pp*NHase and *Pp*NHase‐β‐T_M67C_ at 300 K. e) The RMSF of the β‐subunit of *Pp*NHase and *Pp*NHase‐β‐T_M67C_ at 350 K. f) The ΔRg of *Pp*NHase and *Pp*NHase‐β‐T_M67C_ during MD simulation. g) Substrate access tunnel prediction for both *Pp*NHase and *Pp*NHase‐β‐T_M67C_ under 300 K using CAVER Analyst 2.0.

To get insights into the enhanced catalytic performance of *Pp*NHase‐β‐TM67C, we first constructed a multimeric model of *Pp*NHase‐β‐TM67C using the AlphaFold3 server^[^ ^19^ ^]^ to predict TERM‐M67C multimer structure and LZerD serverfor^[^ ^34^ ^]^ protein‐protein docking of *Pp*NHase to TM67C multimer (**Figure** **5**a). The modeled structure showed that when fused with the hetero‐tetrameric *Pp*NHase, the TERM‐M67C scaffold maintains its fibrous structure, with *Pp*NHase displayed on the outer surface of this fibrous structure (Figure S22, Supporting Information). The outer diameter of this structure is ≈22 nm, consistent with TEM observations of *Pp*NHase‐β‐TM67C fiber. Similar to the previous oligomeric structure of TM67C‐(GGGGS)2‐XynA‐(GGGGS)2‐TM67C, the *Pp*NHase‐β‐TM67C oligomer adopts a helical twist due to the inherent helical structure of the TERM‐M67C core.

Intriguingly, some naturally occurring oligomeric enzymes, such as nitrilases, form left‐handed helices or twists (Figure S23, Supporting Information).^[^ ^35^ ^]^ These hierarchical structures are known to exhibit enhanced thermostability due to inter‐layer interactions, and the concentrated active sites along the long helix potentially contribute to increased enzymatic activity^[^ ^36^, ^37^ ^]^ In addition, it is reported that high‐molecular‐weight NHase (H‐NHase) shows higher stability than its low‐molecular‐weight (L‐NHase) counterpart. The H‐NHase from *Rhodococcus rhodochrous* J1, a 24‐mer metalloenzyme, is widely used for industrial production of acrylamide because of its robustness (Figure S23, Supporting Information).^[^ ^38^ ^]^ However, most reported NHases are tetrameric L‐NHases.^[^ ^39^ ^]^ We speculated that the unique self‐assemble structure in our case may facilitate interactions between interface amino acids of different *Pp*NHase tetrameric units, thereby enhancing the overall integrity and stability of the multimeric complex, mirroring the behavior observed in nitrilases or the H‐NHase.

To validate our hypothesis, we conducted MD simulations of both the WT *Pp*NHase (apo) and *Pp*NHase‐β‐T_M67C_ (apo) at 300 and 350 K. The results showed that during the simulation process, the NHase fused with TERM‐M67C approached each other, creating new contacts between adjacent *Pp*NHase tetramers (Videos S1 and S2, Supporting Information). We further analyzed the trajectories of five adjacent *Pp*NHase tetramers located at the central position of the *Pp*NHase‐β‐T_M67C_ fiber, and the results show that on average, 60.77 hydrogen bonds were formed between the tetrameric interfaces of the five *Pp*NHase tetramers (Figure 5b; Table S3, Supporting Information). These newly introduced interactions may contribute to the stability of the interfaces between different tetramers.

Then we calculated the DCCM of the WT *Pp*NHase and *Pp*NHase‐β‐T_M67C_ at 350 K. The DCCM matrices revealed significant correlations among the *Pp*NHase subunits within the *Pp*NHase‐β‐T_M67C_ pentamers, indicating that the system behaves as a cohesive entity rather than as isolated units during high‐temperature simulations (Figure 5c). Furthermore, by comparing the DCCM matrices of the monomeric *Pp*NHase‐β‐T_M67C_ and *Pp*NHase, we observed that the overall integrity of the NHase component in the monomeric *Pp*NHase‐β‐T_M67C_ is significantly enhanced, which is attributed to the cohesive nature of the multimeric system (Figure 5c; Figure S24, Supporting Information). This result is mainly due to two factors: first, the fusion of TERM‐M67C onto the β‐subunit of NHase enhances interactions within the β‐subunits of NHase; second, the formation of new inter‐protein interfaces within the system contributes to the observed effects. These factors collectively contribute to the improved stability of *Pp*NHase‐β‐T_M67C_.

To further explore the impact of TERM‐M67C on *Pp*NHase‐β‐T_M67C_, we calculated the RMSF of the α and β subunits at 300 and 350 K. The RMSF results indicated that at 350 K, all four subunits of monomeric *Pp*NHase‐β‐T_M67C_ exhibited lower fluctuations than the WT *Pp*NHase (Figure 5d,e). Then, we further explored the impact of protein oligomerization on *Pp*NHase by simulating the pentameric form of *Pp*NHase‐β‐T_M67C_. Like *Pp*NHase‐β‐T_M67C_, the NHase regions closer to the fibril core exhibited greater stability, suggesting that both monomeric and multimeric TERM‐M67C scaffolds can protect guest proteins via polymerization (Figure S25, Supporting Information).

Additionally, we examined the free energy profiles of *Pp*NHase and *Pp*NHase‐β‐T_M67C_ during simulations. The free energy landscape for *Pp*NHase revealed two energy wells, indicating conformational instability at 350 K and reflecting its relatively low stability. Conversely, *Pp*NHase‐β‐T_M67C_ displayed a single energy well, confirming its stable structure at elevated temperatures (Figure S26, Supporting Information). The ΔRg further confirms this phenomenon. The ΔRg of *Pp*NHase‐β‐T_M67C_ is significantly lower than that of *Pp*NHase. Together with previous DCCM results and the protein's free energy analysis, it suggests that *Pp*NHase‐β‐T_M67C_ becomes more stable due to polymerization (Figure 5f).

The formation of new interface interactions between the tetrameric units of *Pp*NHase immobilized on TERM‐M67C might potentially alter the spatial configuration of *Pp*NHase itself. To verify this, we extracted the lowest free energy conformations of the WT *Pp*NHase and the *Pp*NHase tetramer extracted from *Pp*NHase‐β‐T_M67C_ during simulations and performed substrate access tunnel predictions. At 300 K, the predictions showed two potential substrate access tunnels for the WT *Pp*NHase, compared to four substrate access tunnels for *Pp*NHase‐β‐T_M67C_ (Figure 5g). A higher number of substrate access tunnels suggests a higher possibility of substrate and product migration,^[^ ^40^ ^]^ which correlated with the lower *K*_m_ of *Pp*NHase‐β‐T_M67C_ toward the substrate (Table 2; Figure S18, Supporting Information)

### 2.7 TERM‐M67C as a Potential Efficient Platform for Dual‐Enzyme Cascade Reactions

When two enzymes are in close spatial proximity, their cascade reactions can significantly enhance substrate conversion efficiency.^[^ ^41^ ^]^ This proximity allows the product of one enzyme to quickly become the substrate for the other, reducing reaction time and increasing the overall reaction rate.^[^ ^42^ ^]^ To determine the efficiency of an enzyme cascade platform, controllability, and high loading capacity are the most crucial factors. Traditional click chemistry and bio‐orthogonal polymerization are challenging to ensure the controllability of enzyme cascades. Additionally, limited by structural space constraints, achieving a satisfactory level of enzyme loading capacity is difficult. Benefiting from direct genetic fusion, the substrate protein loading capacity of the TERM‐M67C scaffold reaches a complete 100%. Furthermore, TERM‐M67C features two open substrate protein fusion ports (its N‐terminus and C‐terminus), rendering it an efficient cascading platform.

**Figure 6:**
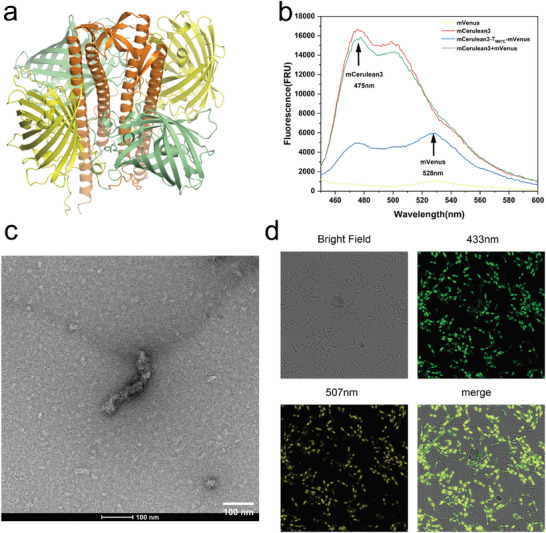
The FRET analysis and the unique cellular localization of mCerulean3‐T_M67C_‐mVenus. a) The model of mCerulean3‐T_M67C_‐mVenus single layer. The mCerulean3 was shown as a green cartoon and the mVenus was shown as a yellow cartoon. The orange part represents the TERM‐M67C. The structure was constructed by AlphaFold3 server. b) Fluorescence intensity analysis of free mVenus, free mCerulean3, mixture of free mVenus and mCerulean3, and mCerulean3‐T_M67C_‐mVenus. An excitation wavelength in the 450–600 nm range and emission at 413 nm was used. The proteins are diluted to qual molar concentrations (0.5 µm). c) Typical configuration of mCerulean3‐T_M67C_‐mVenus observed under TEM, showing a short rod‐like structure. d) Confocal laser microscopy images of *E. coli* expressing mCerulean3‐T_M67C_‐mVenus. Images were taken under a bright field, at 433 nm, and at 507 nm. The merged image combines photos from the two different wavelengths.

Consequently, we fused two monomeric fluorescent proteins, mCerulean3 and mVenus, with emission wavelengths of 475 nm and 528 nm respectively, to the N‐ and C‐termini of the TERM‐M67C protein scaffold linked by (GGGGS)_2_, resulting in mCerulean3‐T_M67C_‐mVenus (**Figure** **6**a; Figure S27, Supporting Information). This design has dual aims: firstly, to assess protein‐protein interactions via FRET, and secondly, to confirm whether TERM‐M67C can still form high molecular weight assemblies when guest proteins are attached at both termini.

The FRET analysis of purified mCerulean3‐T_M67C_‐mVenus indicated that, under an emission wavelength of 475 nm, the donor mCerulean3 exhibited the highest fluorescence intensity individually (16 654 a.u.). with an emission wavelength of 475 nm, the sole donor mCerulean3 displayed its highest fluorescence intensity at 16 654 a.u. In a mixed system with free fluorescent proteins, the donor's fluorescence intensity was 15 810 a.u. However, in the fusion protein mCerulean3‐T_M67C_‐mVenus, at 475 nm, the fluorescence intensity of mCerulean3 dropped from 16 654 to 4657 a.u., while at 528 nm, the fluorescence intensity of mVenus increased from 1036 a.u. to 5973 a.u. (Figure 6b).

In a FRET assay, the greater the fluorescence loss, the closer the donor and receptor are to each other. Therefore, we calculated the FRET efficiency in two different systems. In the direct mixture of free mVenus and mCerulean3, the FRET efficiency was 6%, while in the fusion protein system, the FRET efficiency reached 72% (Table S4, Supporting Information). This notable increase demonstrates a strong FRET signal, suggesting proximity between mCerulean3 (the donor) and mVenus (the acceptor) within the protein scaffold. The high level of fluorescence quenching suggests that the distance between the two fused proteins is optimal for FRET, typically within 1–10 nm.^[^ ^43^ ^]^

Subsequently, we measured the molecular weight of the fusion protein mCerulean3‐T_M67C_‐mVenus using SEC. The results indicated that mCerulean3‐T_M67C_‐mVenus could still form high molecular weight assemblies, with the maximum reaching up to 34‐mer (Figure S28, Supporting Information). TEM images reveal that mCerulean3‐T_M67C_‐mVenus still assembles into short rod‐like structures (Fig 6c). Additionally, laser confocal microscopy images of mCerulean3‐T_M67C_‐mVenus within *Escherichia coli* cells revealed an intriguing pattern: after in vivo assembly, mCerulean3‐T_M67C_‐mVenus aggregated at both poles of the cell, whereas this phenomenon did not occur in *E. coli* expressing only the fluorescent proteins. (Figure 6d; Figure S29, Supporting Information). This protein aggregation is similar to membrane‐less organelles mediated by liquid‐liquid phase separation which needs to be investigated in further studies. Many studies have shown that such aggregation can enhance intracellular enzyme catalytic activity.^[^ ^44^ ^]^ These findings support the hypothesis that TERM‐M67C can bring the two fluorescent proteins into proximity to facilitate efficient energy transfer.

## 3 Conclusion

In this work, we have successfully developed a protein scaffold termed TERM‐M67C by designing a disulfide bond within a self‐assembling protein. This scaffold offers significant potential and practical value by enabling one‐step enzyme polymerization, enhancing protein catalytic efficiency, and accommodating a wide range of additional functionalities. The TERM‐M67C scaffold exhibits exceptional compatibility with guest proteins, regardless of whether they are monomeric, multimeric, or have a complex maturation mechanism. Furthermore, the fusion with the TERM‐M67C scaffold not only enhances the stability of the guest proteins but also increases their catalytic activity. This improvement is attributed to the extra protection offered by the scaffold and the higher local concentration of the proteins. Additionally, when TERM‐M67C accommodates two fluorescent proteins, it can facilitate strong Förster resonance energy transfer (FRET), indicating its potential as an efficient enzyme cascade platform.

## 4 Experimental Section

### Raw Material Preparation

*E. coli* Shuffle T7 B was purchased from Shanghai Weidi Biology. All chemical reagents were purchased from China Pharmaceutical Group. Gene synthesis services are provided by Wuxi Tianlin Biotechnology Co., Ltd. DNA polymerase, restriction enzymes, etc. were purchased from Takara. All protein purification columns were purchased from GE Healthcare.

### Reproducibility and Replicability

All experiments were repeated three times or more to ensure the accuracy and reproducibility of the results. Representative data was selected for presentation, and statistical testing was based on three repeated experiments.

### Cultivation and Expression Conditions

*E. coli* Shuffle T7 and BL21(DE3) were used as expression hosts for target gene expression. Genes encoding TERM, TERM‐M67C, *Pp*NHase, mCerulean3, mVenus, *Pp*NHase‐β‐T_M67C_, T_M67C_‐XynA‐T_M67C_, mCerulean3‐T_M67C_‐mVenus were cloned into the vector pET‐24a (+) after codon optimization and transformed into *E. coli* by heat shock. Overnight cultures were inoculated at a 1% inoculum into 500 mL of 2 × YT medium supplemented with a final concentration of 50 µg mL^−1^ kanamycin. Cells were grown at 37 °C, 200 rpm until OD_600_ reached 0.6–0.8, then induced with a final concentration of 0.4 mm IPTG. For NHase induction, an additional final concentration of 0.1 g L^−1^ Co^2+^ was added to meet its active site maturation requirements, and cultivation continued at 25 °C, 200 rpm for 16 h. Cells were collected by centrifugation at 13 000 rpm for 5 min, then resuspended in 10 mm KPB buffer (10 mm potassium dihydrogen phosphate, 10 mm dipotassium phosphate).

### Determination of Disulfide Bond Content in Proteins

Disulfide bonds were determined using a slightly modified version of the Ellman method.^[^ ^22^ ^]^ Briefly, the purified protein was dissolved in Tris‐HCl buffer (pH 8.0) to a final concentration of 0.6 mg mL^−1^. Then, 100 µL of the protein solution was mixed with 100 µL of reaction buffer (50 mm KH_2_PO_4_‐K_2_HPO_4_, 10 mm ethylenediaminetetraacetic acid (EDTA), 0.6 m KCl, 10 mm DTT, pH 7.0). 2 µL of 0.1% 5,5′‐dinitrobis (2‐nitrobenzoic acid) (DTNB) was then added to the mixture and incubated at 40 °C for 30 min. Absorbance was measured at 412 nm to calculate the total sulfhydryl (SH) groups. Reactive SH groups were determined by incubating the reaction mixtures under conditions without DTT at 37 °C for 60 min. Standard curves were generated using different concentrations of cysteine.

### Protein Purification

The resuspended cells were sonicated for 30 min, and the resulting lysate was centrifuged at 13 000 rpm for 20 min. The crude enzyme solution was filtered through a 0.22 µm filter membrane and loaded onto a 5 mL StrepTrap XT column, with the entire process carried out on an AKTA purifier. The binding buffer used was 10 mm KPB, and the elution buffer contained an additional 50 mm D‐biotin. The eluted proteins were collected, and their concentrations were measured using the Bradford method before being stored in a −80 °C freezer for future use.^[^ ^45^ ^]^ All SDS‐PAGE (15% Tris‐Glycine Page) analyses of pure enzymes were performed with equal volumes (10 µL) and equal molar concentrations (5 µm) for loading.

### Förster Resonance Energy Transfer (FRET) Analysis

Set up control samples: meCerulean3, mVenus, and mCerulean3‐T_M67C_‐mVenus. Prepare sample solutions with a concentration of 0.5 µm. Take 100 µL of each sample and place it in a black, enzyme‐labeled 96‐well plate for detection. The excitation wavelength was set to 413 nm, and the emission wavelengths were scanned from 450 to 600 nm, recording each measurement at 1 nm intervals. Process and plot the detection data using Origin software. The efficiency is calculated by *E*_T_ = 1− *F*_DA_/*F*_D_.^[^ ^46^ ^]^ F_DA_ represents the fluorescence intensity of the donor in the presence of the acceptor, F_D_ was the fluorescence intensity of the donor when the acceptor was absent, and *E*_T_ denotes the efficiency of FRET.

### Characterization of the Molecular Weight of TERM‐M67C Scaffold and Fusion Protein

HiTrap S400HR gel filtration column was used to determine the molecular weight of TERM, TERM‐M67C, and mCerulean3‐T_M67C_‐mVenus. HiTrap S400HR is specifically designed for detecting high molecular weight proteins, and the buffer solution used is PBS at pH 7.4 containing 150 mm NaCl. A volume of 5 mL of purified protein was loaded onto the column for detection.

To assess whether the fusion proteins T_M67C_‐XynA‐T_M67C_ and *Pp*NHase‐β‐T_M67C_ form oligomers, the Superdex Increase GL 10/300 gel filtration column was utilized, which detects proteins larger than 600 kDa at an elution volume of ≈10 mL. All gel filtration columns and standard protein samples used in this experiment were purchased from Cytiva.

### Specific Enzyme Activity and Kinetic Parameters Determination

The enzymatic activity of XynA was defined as the amount of enzyme required to produce 1 µm of product (xylose) per minute. The total reaction volume for determining enzyme activity was 0.8 mL, consisting of 10 µL of 1 µm enzyme, 90 µL of 0.1 m sodium citrate‐phosphate buffer at pH 4.0, and 100 µL of 10 g L^−1^ xylan as the substrate. The reaction was conducted at 50 °C for 5 min, after which 600 µL of 3,5‐Dinitrosalicylic acid solution was added to terminate the reaction. The mixture was then heated at 100 °C for 10 min, and the absorbance was measured at 540 nm. For the determination of kinetic constants of XynA and T_M67C_‐XynA‐T_M67C_, the reaction conditions remained the same, except the reaction time was shortened to 3 min, and the xylan concentration was varied between 0–134 mm.

The nitrile hydratase (NHase) activity was defined as the amount of enzyme required to produce 1 µm of product (amide) per unit of minute. The total reaction volume for determining enzyme activity was 1 mL, which consists of 10 µL of 0.5 µm enzyme solution and 490 µL of 100 mm acrylonitrile as the substrate. The reaction was allowed to proceed for 5 min before being terminated by the addition of 500 µL of 0.1 m phosphoric acid. The product was then detected using HPLC, with a reverse‐phase C18 column, an acetonitrile‐to‐water ratio of 1:2 as the mobile phase (pH 2.9), a flow rate of 0.6 mL min^−1^, and detection at a wavelength of 215 nm over a collection time of 10 min. For the determination of the kinetic constants of *Pp*NHase and *Pp*NHase‐β‐T_M67C_, the same detection method is used, but the reaction time is reduced to 1 min, and the acrylonitrile concentration varies between 0–600 mm.

### Whole‐Cell Catalytic Production of Acrylamide

To accurately measure the cell biomass, a spectrophotometer was used at OD_600_. First, 10 mL of bacterial culture was transferred to a centrifuge tube and centrifuged at 13 000 rpm for 5 min to obtain the cell pellet. The cell pellet was then resuspended in 1 mL of 0.01 m KPB buffer (pH 7.4) and thoroughly mixed by shaking to ensure uniform distribution of cells, effectively concentrating the cells. An appropriate volume of the resuspended cell solution was taken and diluted to ensure that the OD_600_ of the diluted solution was between 0.2 and 0.8. The OD_600_ of the diluted solution was measured using a spectrophotometer. The actual OD of the resuspended cell solution was calculated based on the dilution factor using the formula: OD of resuspended cells = OD_600_ of diluted solution × dilution factor. After obtaining the accurate OD value, the resuspended cell solution was re‐diluted to an OD_600_ of 9.3. Finally, 23 mL of the bacterial culture with an OD600 of 9.3 was mixed with 23 mL of acrylonitrile substrate to initiate the catalytic reaction. The reaction involves a single‐step catalytic process and operates without temperature control. Samples are collected from the system at intervals of 10, 30, 60, and 120 min. Each sampling event involves adding 100 µL of the sample to 900 µL of 0.1 m phosphoric acid to halt the reaction, followed by a 1000‐fold dilution for subsequent liquid phase detection. Standard curves were generated based on the peak areas corresponding to acrylamide concentrations of 0.5, 1, 2, 5, and 10 mm.

### Transmission Electron Microscope Analysis

The negative staining protocols for Transmission Electron Microscope (TEM) involve specific steps for sample preparation and observation. For negative staining, the copper grid was first subjected to hydrophilic treatment (PELCO easiGlow) and then picked up with self‐locking forceps. The sample was transferred onto the grid with a pipette, and after 1 min, the excess liquid was removed with filter paper. A uranyl acetate staining solution was then applied to the grid with a pipette, and the excess liquid was again absorbed after 1 min. The sample was left to dry naturally before being observed and photographed under a Thermo Fisher Talos F200C transmission electron microscope.

### Molecular Dynamic Simulation

The structures of the proteins TERM‐M67C T_M67C_‐_(GGGGS)2_‐XynA‐_(GGGGS)2_‐T_M67C_ were predicted and characterized using AlphaFold Serve.^[^ ^19^ ^]^ Based on the scoring function and confidence score of the server, the best protein model was selected. The structure of *Pp*NHase‐T_M67C_‐β was determined using the combined approach of AlphaFold3 server prediction of TERM scaffold predictions and *Pp*NHase docking to scaffold using the LZerD server.^[^ ^34^ ^]^ The structure of XynA (PDB ID:6QE8), γ‐PFD (PDB ID:6VY1), and *Pp*NHase (PDB ID:3QXE) were crystal structures downloaded from Protein Data Bank. The molecular docking study was conducted using the Ligand Docking module in the Schrödinger software suite. This process strictly adheres to the standard protocols of molecular simulation, optimizing the binding modes between ligands and receptors to predict their interactions and binding affinities.^[^ ^47^ ^]^ Subsequently, MD simulations were performed using GPU GROMACS 2023.2,^[^ ^48^ ^]^ employing the Amber 14SB force field. Protonation states were calculated using PROPKA.^[^ ^49^ ^]^ Simulations were conducted at 300 K and 350 K under atmospheric pressure for a total of 100 ns, and a time step of 2 fs. Electrostatic interactions were calculated using periodic boundary conditions (PBC) and the Particle Mesh Ewald (PME) method.^[^ ^50^ ^]^ The proteins were solved in a TIP3P water box with a minimum boundary distance of 10 Å, and 0.15 m NaCl ions were added to neutralize the system. Temperature and pressure were controlled using the C‐rescale and V‐rescale algorithms, respectively. RMSD and RMSF analyses were carried out using the GROMACS analysis suite, while the binding free energies of the tetrameric proteins were calculated via gmx MMPBSA.^[^ ^29^ ^]^ Visualizations were generated with PyMOL and VMD. The analysis of the NHase substrate channel was performed using Caver 3.0.3.^[^ ^51^ ^]^ The prediction of the substrate channel was conducted by extracting the lowest free energy conformation from the simulated trajectory.

## Conflict of Interest

The authors declare no conflict of interest.

## Author Contributions

Y.M. and L.P. contributed equally to this work. Y.M., Z.C., and Z.Z. conceived the project and wrote the paper. Y.M., T.W., Z.C., L.H., H.G., R.G., Z.L., and Y.X. designed and performed all the experiments. Y.M., Z.C., and L.P. analyzed the results.

## Supporting information



Supporting Information

Supplemental Video 1

Supplemental Video 2

## Data Availability

The data that support the findings of this study are available in the supplementary material of this article.;
