# Supplementary material for: A Versatile Protein Scaffold Engineered for the Hierarchical Assembly of Robust and Highly Active Enzymes
Source: Adv Sci (Weinh). 2025 Feb 22;12(15):2500405. doi: 10.1002/advs.202500405 (PMC12005783; doi:10.1002/advs.202500405)
Supplement: Supplementary file 1 — Supporting Information [file ADVS-12-2500405-s002.docx]

**Supplementary Information**

For

**A Versatile Protein Scaffold Engineered for the Hierarchical Assembly of Robust and Highly Active Enzymes**

Yiwei Meng^1#^, Lukasz Peplowski^2#^, Tong Wu^1^, Heng Gong^1^, Ran Gu^1^, Laichuang Han^1^, YuanYuan Xia^1^, Zhongmei Liu^1^, Zhemin Zhou^1, 3^, Zhongyi Cheng^1*^

^1^ Key Laboratory of Industrial Biotechnology (Ministry of Education), School of Biotechnology, Jiangnan University, Wuxi, Jiangsu, China

^2^ Institute of Physics, Faculty of Physics, Astronomy and Informatics, Nicolaus Copernicus University in Torun, Grudziadzka 5, 87-100 Torun, Poland

^3^ Jiangnan University (Rugao) Food Biotechnology Research Institute, Rugao, Jiangsu, China

^#^These authors contributed equally to this work

*corresponding authors：

Zhongyi Cheng,

School of Biotechnology, Jiangnan University

Wuxi, Jiangsu, 214122, China

E-mail: [zyCheng@jiangnan.edu.cn](mailto:zyCheng@jiangnan.edu.cn)

**Table of Contents**

Supplementary Tables…………………………………………………..3-7

Supplementary Figures ……………………………………………….8-36

**Tables**

**Table S1.** The potential inter-chain disulfid bond predicted by Disufide by Design 2.

| **Residue pairs** | **Original** | **Mutated** |
| --- | --- | --- |
| Chain A:67 - Chain B:67 | MET-MET | CYS-CYS |
| Chain A:66 - Chain B:71 | GLU-LYS | CYS-CYS |
| Chain A:61 - Chain B:39 | SER-SER | CYS-CYS |

**Table S2.** The number of disulfide bonds in TERM-M67C was determined by the Ellman method.

| Protein | Concentration | Free -SH | Total -SH | Disulfide bond |
| --- | --- | --- | --- | --- |
| TERM-M67C | 1μM | 2.21μM | 2.99μM | 0.39μM |

**Table S3**. The new protein-protein interactions that formed between the tetrameric interfaces of *Pp*NHase-β-T_M67C_ tetrameric units during simulations at the lowest free energy conformations.

| Set1 Residues | Set2 Residues | Distance(Å) | Specific Interactions | HB | Salt Bridges |
| --- | --- | --- | --- | --- | --- |
| B:Arg 242 | C:Glu 351 | 2.1 | 3x hb, 2x salt bridge to C:Glu 351 | 3 | 2 |
| B:Leu 285 | C:Ala 313 | 1.8 | 2x hb to C:Ala 313 | 2 | 0 |
| B:Ala 313 | A:Leu 285 | 1.8 | 2x hb to A:Leu 285 | 2 | 0 |
| B:Ala 313 | C:Leu 285 | 1.9 | 2x hb to C:Leu 285 | 2 | 0 |
| B:Leu 285 | A:Ala 313 | 2 | 2x hb to A:Ala 313 | 2 | 0 |
| B:Arg 242 | A:Glu 351 | 2.1 | 2x hb, 1x salt bridge to A:Glu 351 | 2 | 1 |
| B:Glu 249 | C:Arg 252 | 2.1 | 2x hb to C:Arg 252 | 2 | 0 |
| B:Glu 249 | C:Tyr 347 | 1.8 | 1x hb to C:Tyr 347 | 1 | 0 |
| B:Gln 198 | A:Ala 366 | 1.9 | 1x hb to A:Ala 366 | 1 | 0 |
| B:Gln 293 | A:Ala 311 | 1.9 | 1x hb to A:Ala 311 | 1 | 0 |
| B:Gln 359 | C:Ser 224 | 1.9 | 1x hb to C:Ser 224 | 1 | 0 |
| B:Ser 361 | A:Gln 358 | 2 | 1x hb to A:Gln 358 | 1 | 0 |
| B:Ala 311 | C:Gln 293 | 2 | 1x hb to C:Gln 293 | 1 | 0 |
| B:Tyr 347 | A:Glu 249 | 2 | 1x hb to A:Glu 249 | 1 | 0 |
| B:Thr 283 | C:Glu 315 | 2 | 1x hb to C:Glu 315 | 1 | 0 |
| B:Arg 266 | A:Glu 254 | 2 | 1x hb to A:Glu 254 | 1 | 0 |
| B:Glu 328 | A:Ser 274 | 2 | 1x hb to A:Ser 274 | 1 | 0 |
| B:Ser 224 | A:Glu 362 | 2 | 1x hb to A:Glu 362 | 1 | 0 |
| B:Gln 247 | C:Arg 336 | 2 | 1x hb to C:Arg 336 | 1 | 0 |
| B:Ser 361 | C:Glu 362 | 2 | 1x hb to C:Glu 362 | 1 | 0 |
| B:Arg 336 | A:Gln 247 | 2 | 1x hb to A:Gln 247 | 1 | 0 |
| B:Gly 126 | A:Glu 176 | 2 | 1x hb to A:Glu 176 | 1 | 0 |
| B:Gln 341 | A:Glu 239 | 2.1 | 1x hb to A:Glu 239 | 1 | 0 |
| B:Arg 336 | C:Gln 247 | 2.1 | 1x hb to C:Gln 247 | 1 | 0 |
| B:Gly 221 | A:Asp 352 | 2.1 | 1x hb to A:Asp 352 | 1 | 0 |
| B:Lys 178 | A:Asn 157 | 2.1 | 1x hb to A:Asn 157 | 1 | 0 |
| B:Pro 287 | C:Ala 312 | 2.1 | 1x hb to C:Ala 312 | 1 | 0 |
| B:Asp135 | A:Lys 349 | 2.1 | 1x hb, 1x salt bridge to A:Lys 349 | 1 | 1 |
| B:Ala 270 | A:Lys 331 | 2.1 | 1x hb to A:Lys 331 | 1 | 0 |
| B:Gln 247 | A:Arg 336 | 2.2 | 1x hb to A:Arg 336 | 1 | 0 |
| B:Gln 198 | A:Glu 370 | 2.2 | 1x hb to A:Glu 370 | 1 | 0 |
| B:Asp259 | A:Arg 257 | 2.2 | 1x hb to A:Arg 257 | 1 | 0 |
| B:Arg 257 | A:Asp 259 | 2.2 | 1x hb, 1x salt bridge to A:Asp 259 | 1 | 1 |
| B:Gly 222 | A:Asp 352 | 2.2 | 1x hb to A:Asp 352 | 1 | 0 |
| B:Glu 362 | A:Lys 365 | 2.2 | 1x hb, 1x salt bridge to A:Lys 365 | 1 | 1 |
| B:Ala 310 | C:Gln 267 | 2.2 | 1x hb to C:Gln 267 | 1 | 0 |
| B:Ala 311 | A:Gln 293 | 2.2 | 1x hb to A:Gln 293 | 1 | 0 |
| B:Glu 315 | C:Thr 283 | 2.2 | 1x hb to C:Thr 283 | 1 | 0 |
| B:Asn238 | A:Glu 351 | 2.2 | 1x hb to A:Glu 351 | 1 | 0 |
| B:Glu 351 | A:Asn 238 | 2.2 | 1x hb to A:Asn 238 | 1 | 0 |
| B:Glu 254 | A:Arg 266 | 2.3 | 1x hb, 1x salt bridge to A:Arg 266 | 1 | 1 |
| B:Ala 309 | C:Thr 271 | 2.3 | 1x hb to C:Thr 271 | 1 | 0 |
| B:Gln 247 | A:Leu 333 | 2.3 | 1x hb to A:Leu 333 | 1 | 0 |
| B:Thr 271 | A:Ala 309 | 2.3 | 1x hb to A:Ala 309 | 1 | 0 |
| B:Glu 254 | C:Arg 266 | 2.3 | 1x hb to C:Arg 266 | 1 | 0 |
| B:Glu 351 | C:Arg 242 | 2.3 | 1x hb, 1x salt bridge to C:Arg 242 | 1 | 1 |
| B:Lys 136 | A:Glu 356 | 2.3 | 1x hb, 1x salt bridge to A:Glu 356 | 1 | 1 |
| B:Ala 375 | A:Arg 161 | 2.3 | 1x hb to A:Arg 161 | 1 | 0 |
| B:Ser 274 | A:Lys 331 | 2.4 | 1x hb to A:Lys 331 | 1 | 0 |

**Table S4.** The fluorescence intensity of mixture of free mVenus and mCerulean3 and mCerulean3-T_M67C_-mVenus.

| Protein | F_DA_^a^/F_D_^b^ | E_T_^c^=1- F_DA_/F_D_ |
| --- | --- | --- |
| mCerulean3+mVenus | 0.28 | 0.72 |
| mCerulean3-T_M67C_-mVenus | 0.94 | 0.06 |

^a^ the fluorescence intensity of the donor in the presence of the acceptor

^b^ the fluorescence intensity of the donor when the acceptor is absent

^c^ the efficiency of FRET

**Figures**

**
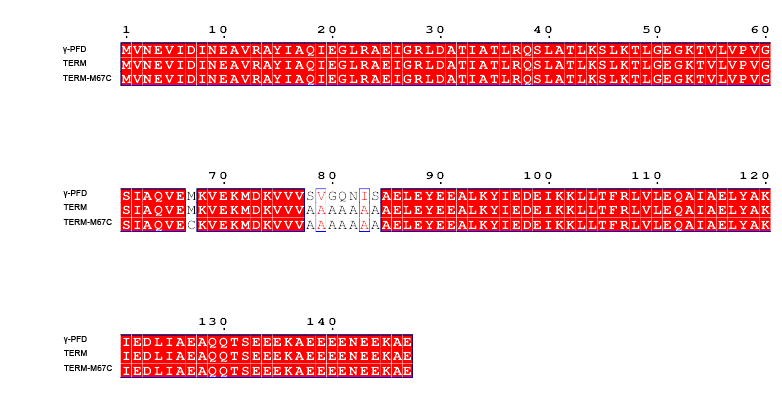
**

**Figure S1.** The sequence alignment of γ-PFD, TERM and TERM-M67C.

**
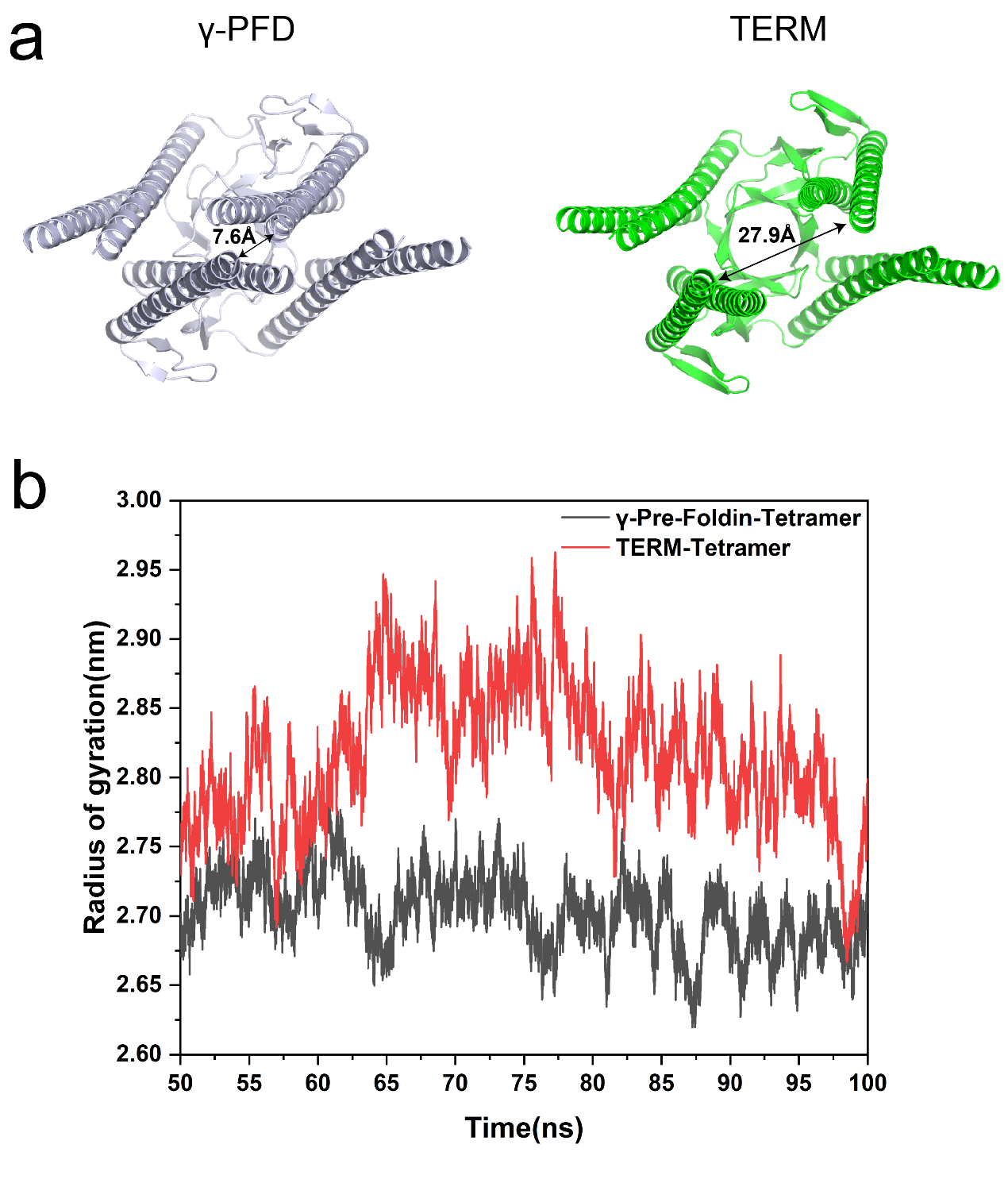
**

**Figure S2.** The radius of gyration (Rg) of γ-PFD and TERM during 100 ns MD simulation. A larger Rg indicates a lower protein density.

**Figure S3.** Determination of the disulfide bond content in proteins using the Ellman method. The standard curve is established with the following concentrations of cysteine: 0, 2, 4, 6, 8, 10, 20, 30, 40, 50, and 60 μM.


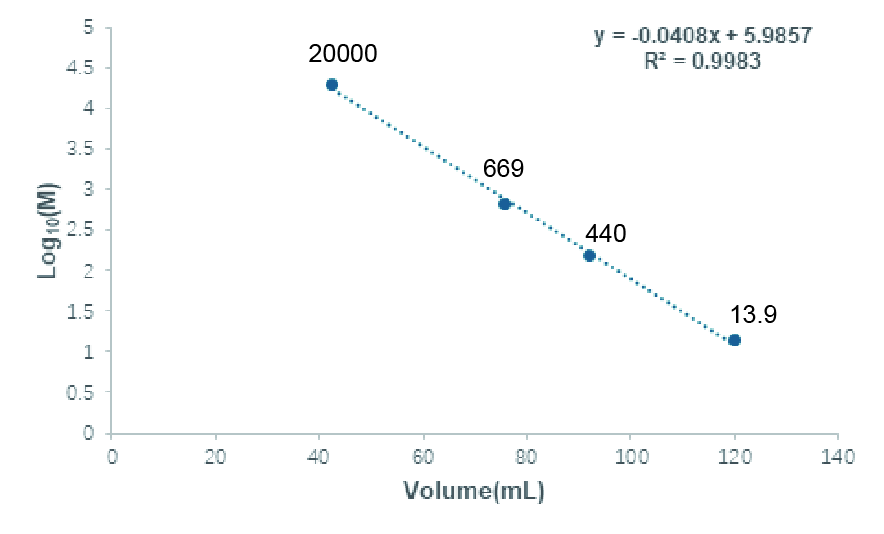


**
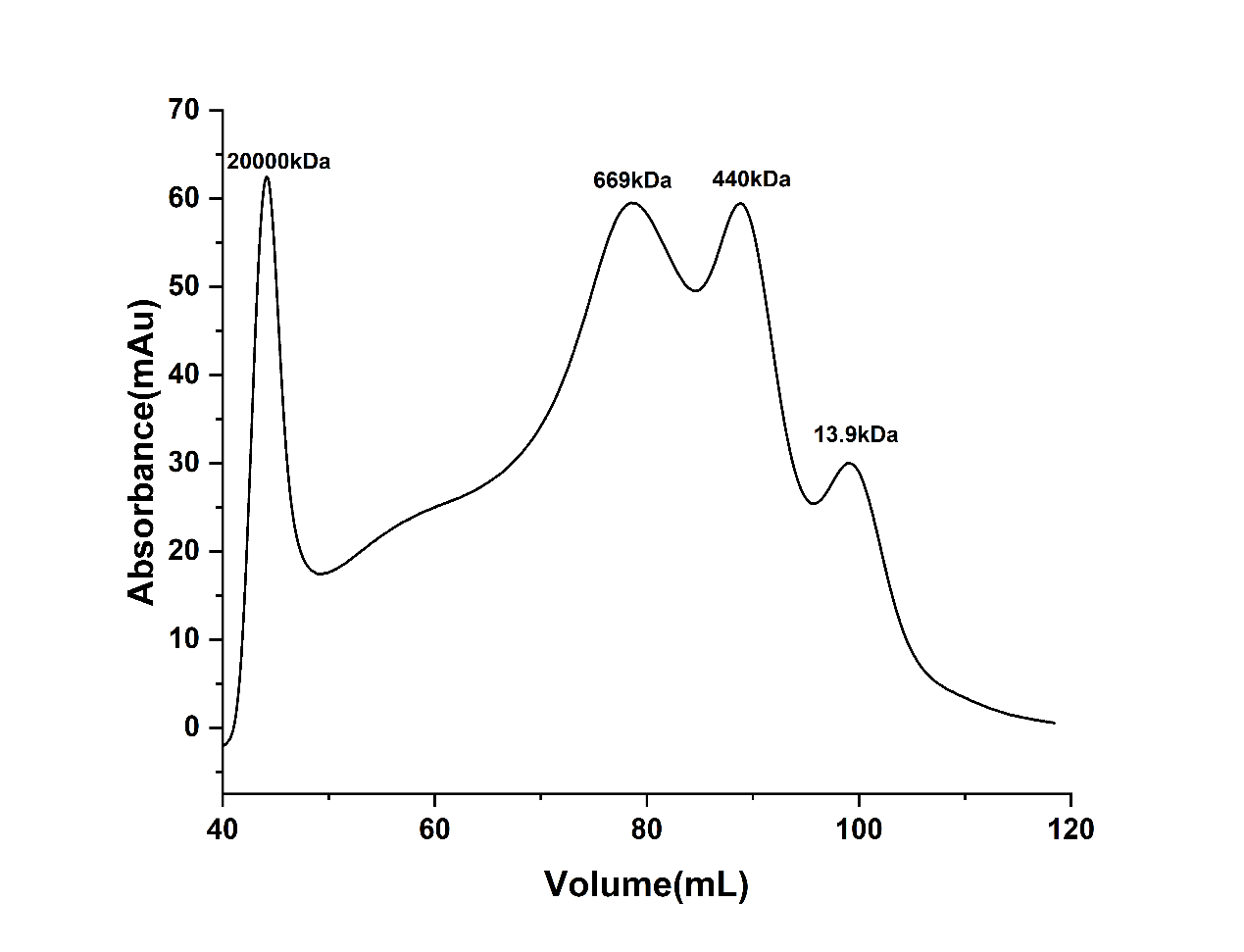
**

**Figure S4.** The standard curve of HiTrap S400HR gel filtration column for determination of the molecular mass and structures of the proteins in this study. The molecular weight of marker proteins used for gel filtration: 20000, 669, 440,13.9 kDa.


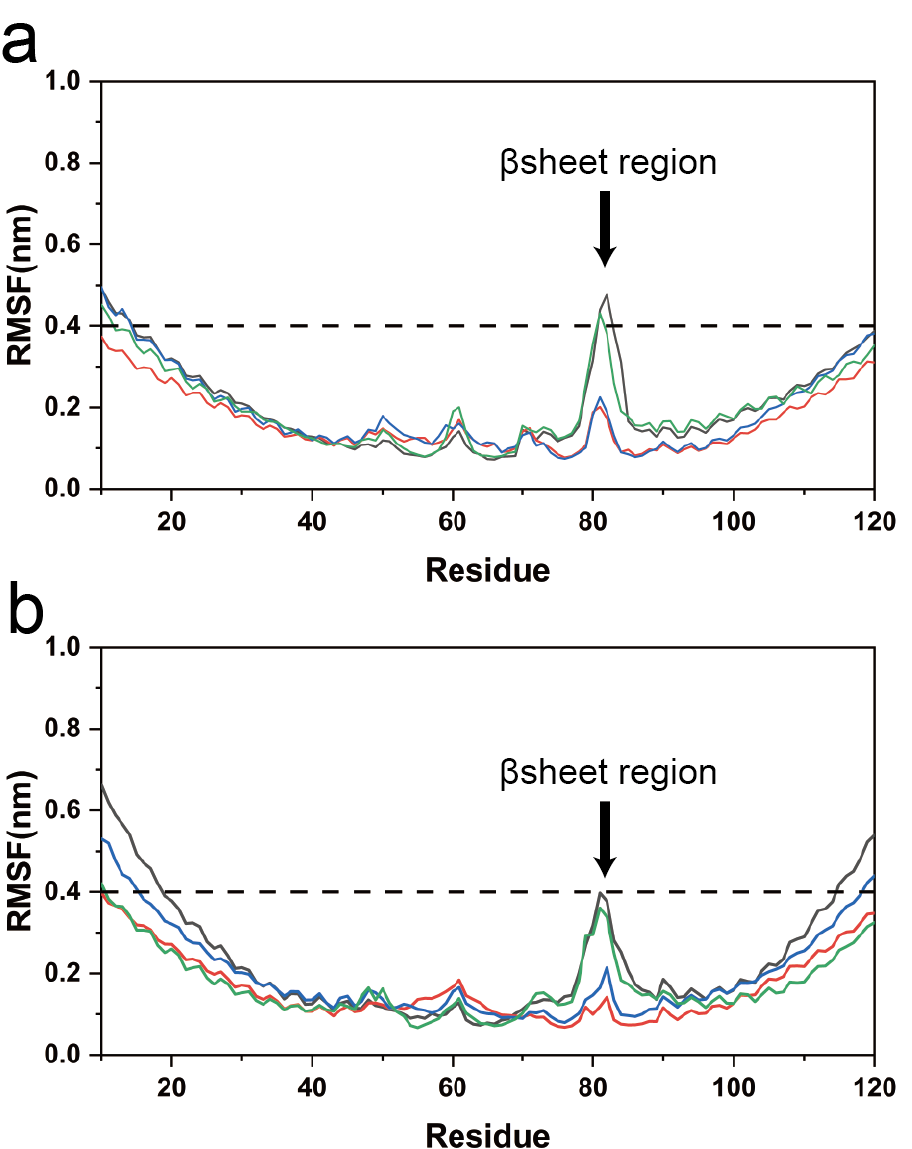


**Figure S5.** The RMSF of TERM and TERM-M67C after 100 ns MD simulation. a) RMSF of TERM under 350K. b) RMSF of TERM-M67C under 350K.


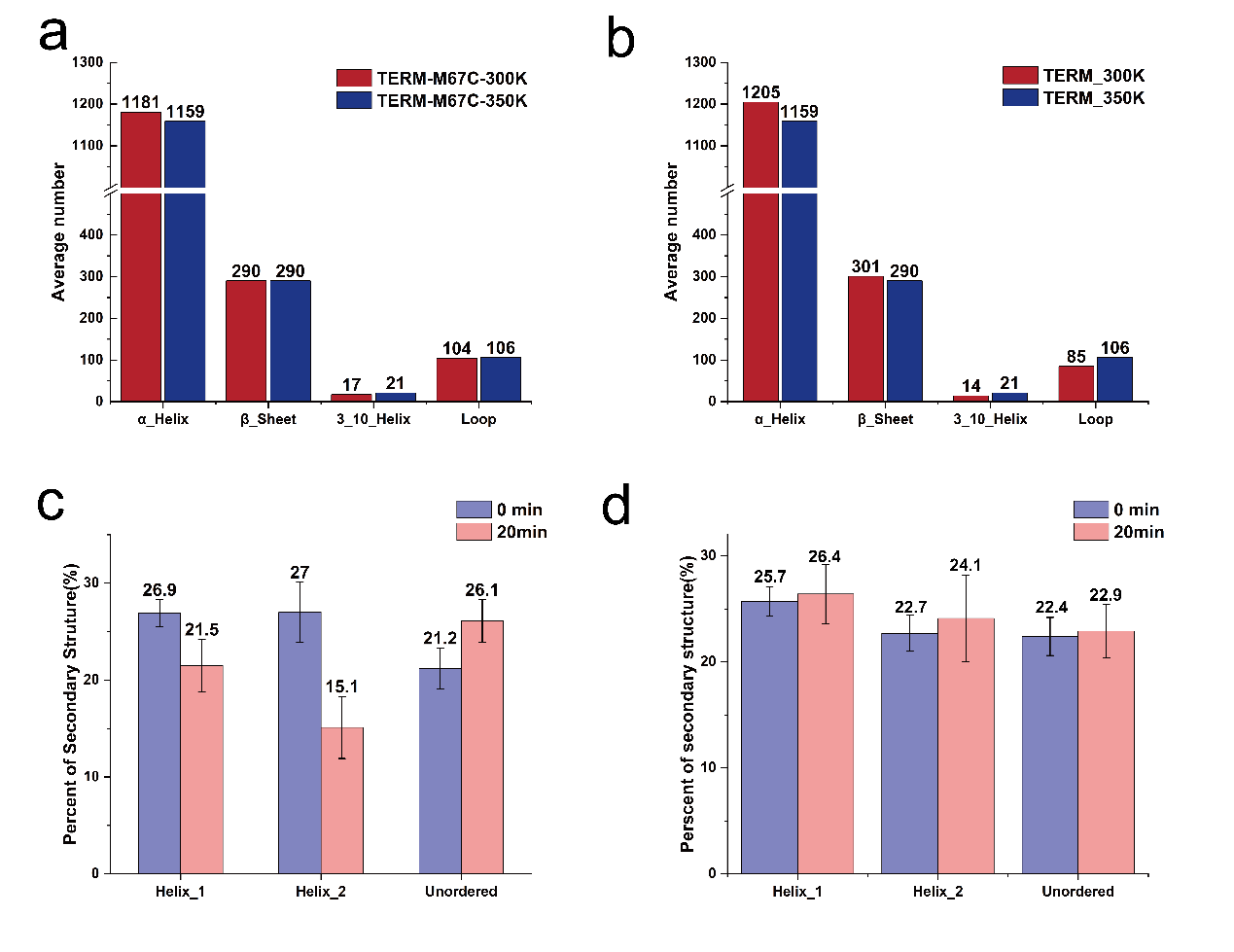


**Figure S6.** The secondary structure change of TERM and TERM-M67C. The plot displays the average occurrence of various secondary structures throughout the simulation. a) TERM 12-mer b) TERM-M67C 12-mer c) The percent of secondary structure of TERM WT before and after 80℃ treatments. d) The percent of secondary structure of TERM-M67C before and after 80℃ treatments.


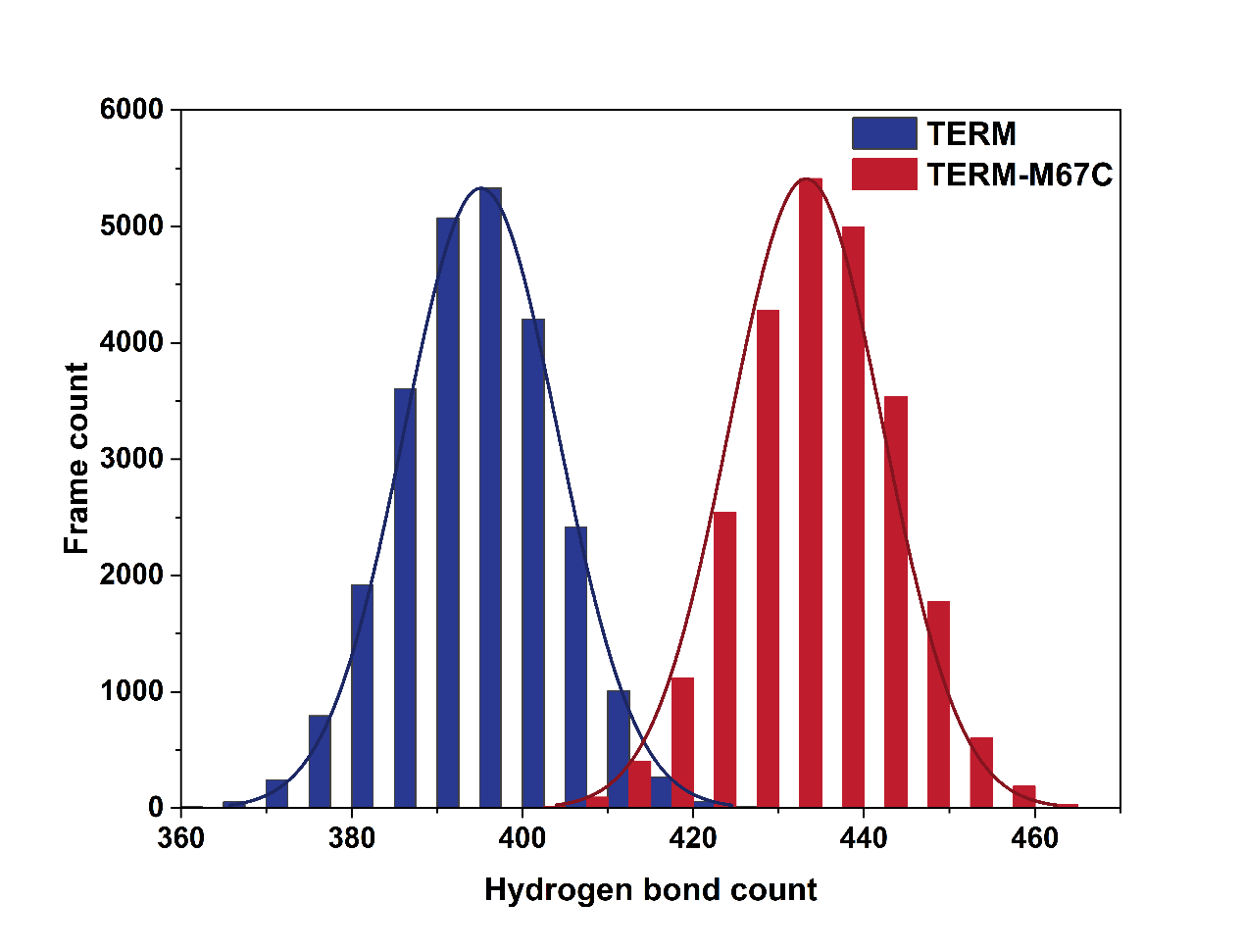


**Figure S7.** Total number of hydrogen bonds in TERM (10-mers) and TERM-M67C (10-mers) under 350K during 100 ns MD simulation.


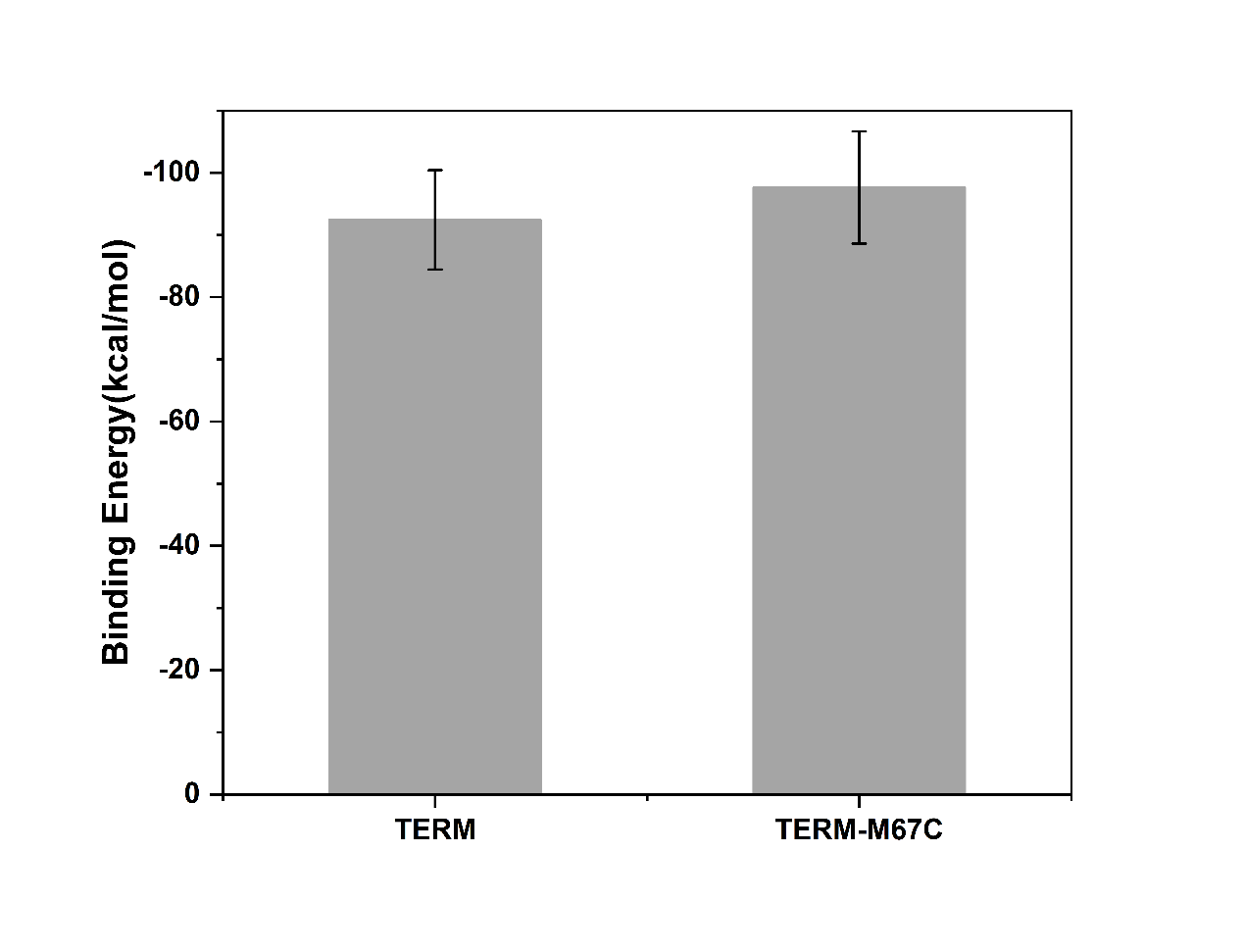


**Figure S8.** The binding free energy between the two adjacent layers of the WT TERM and the mutant TERM-M67C under 350K. The binding energy was calculated by MM/PBSA.


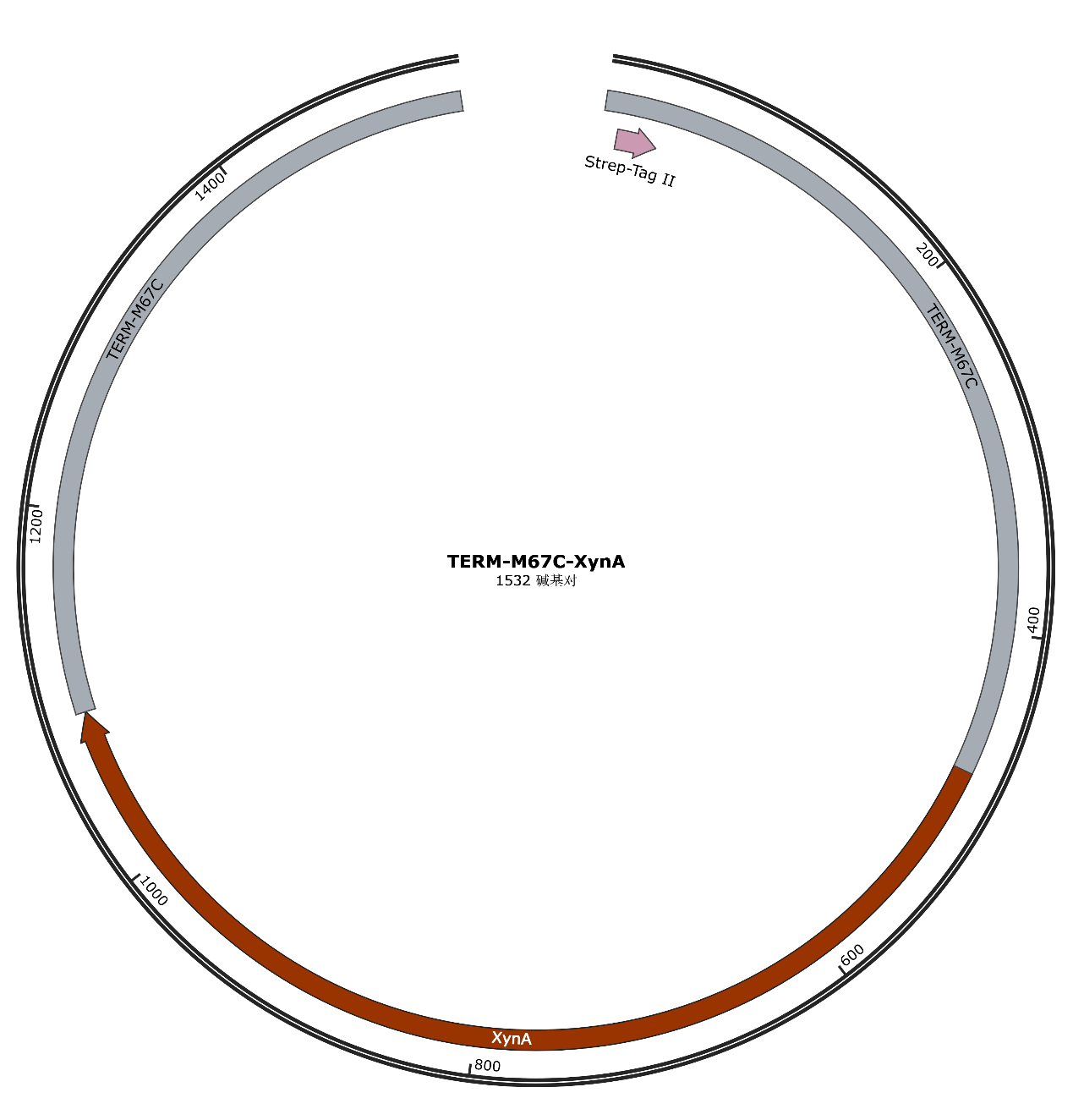


**Figure S9.** Diagram of the Construction Approach for TERM-M67C-XynA-M67C-TERM. The two TERM-M67C are directly fused to the N-terminus and C-terminus of XynA, respectively.

**
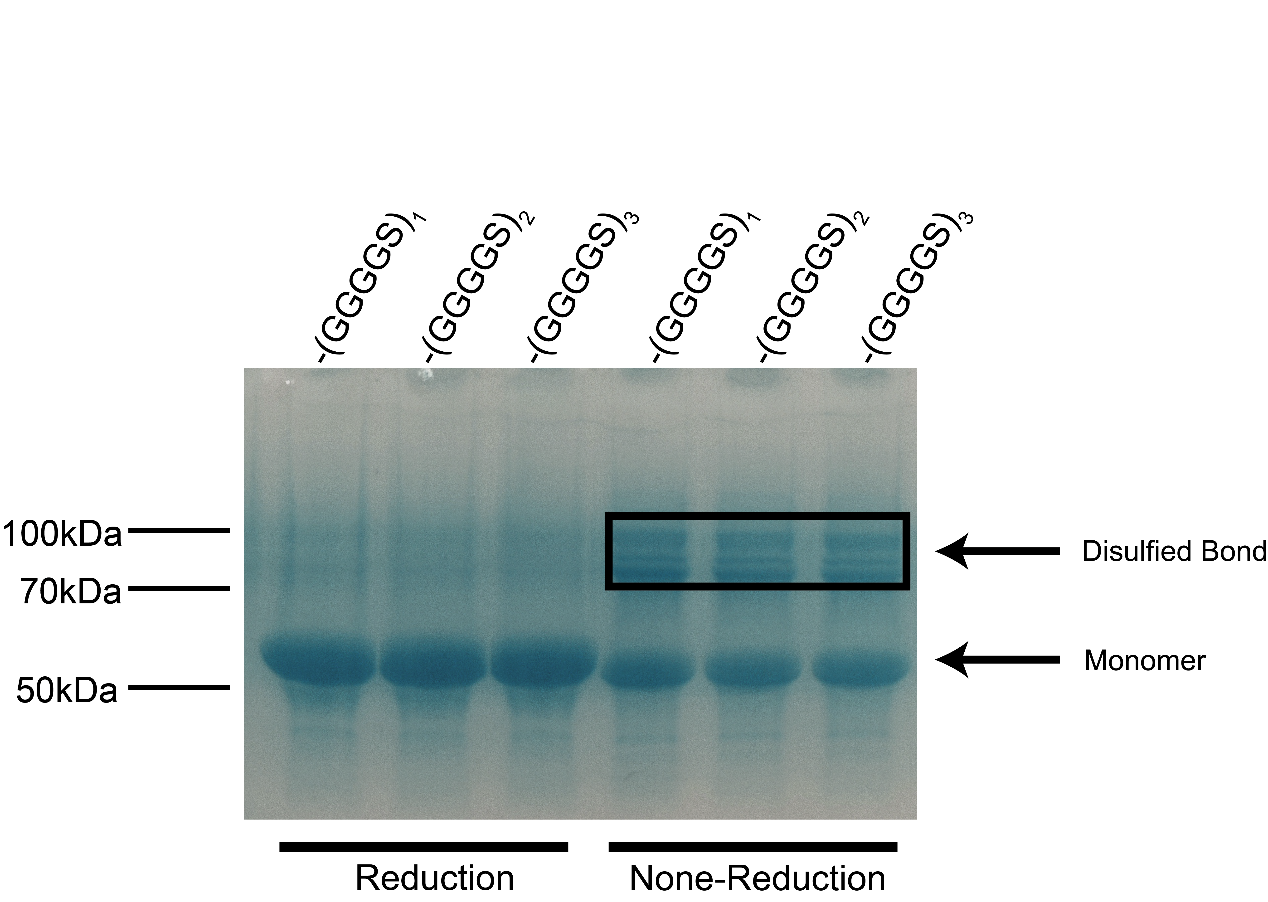
**

**Figure S10.** The SDS-PAGE analysis of T_M67C_-XynA-T_M67C_ with different length of linkers (5μM) under reducing (R) and non-reducing (NR) conditions. β-Mercaptoethanol was used as the reducing agent. The molecular weight of fusion protein is 54kDa. ((GGGGS)_n_ = T_M67C_-_(GGGGS)n_-XynA-_(GGGGS)n_-T_M67C_)


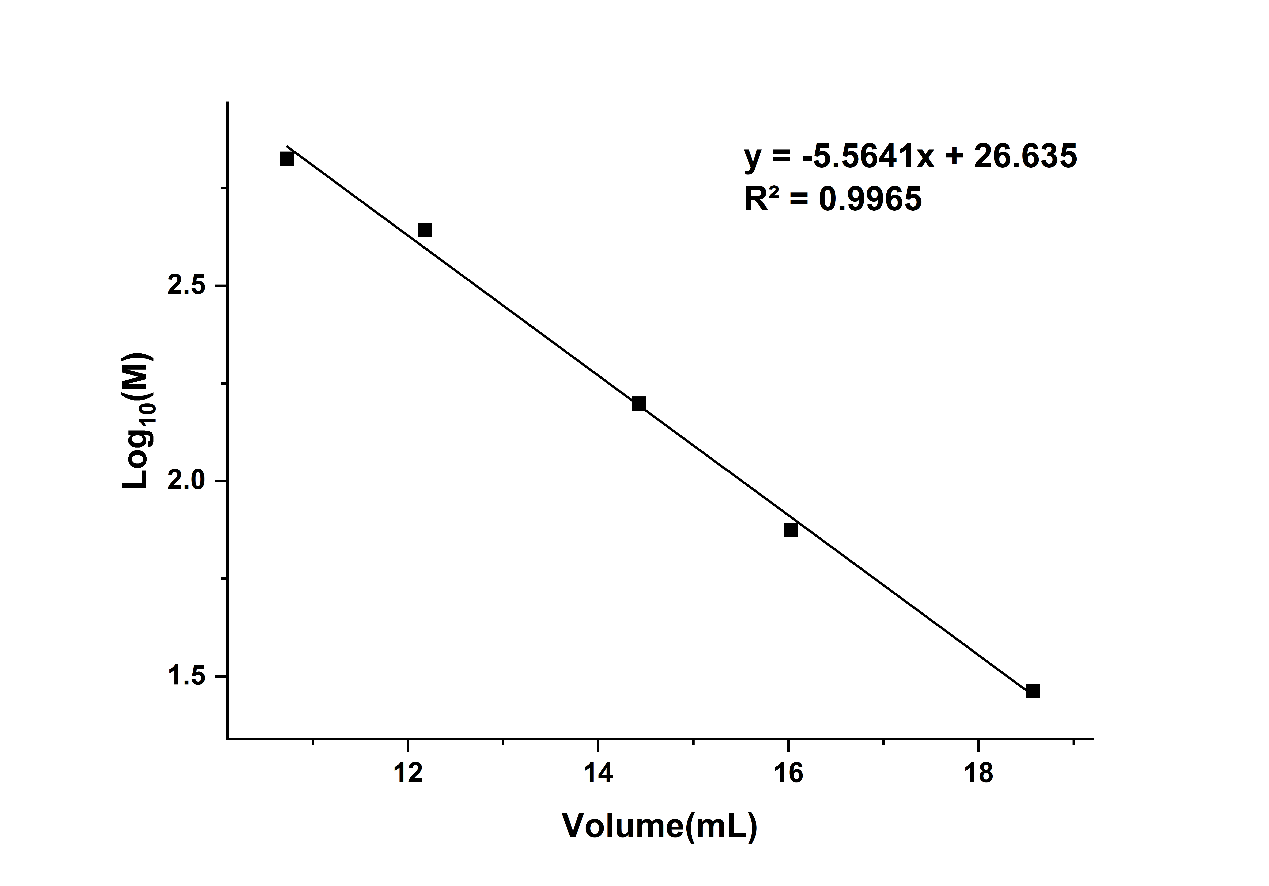


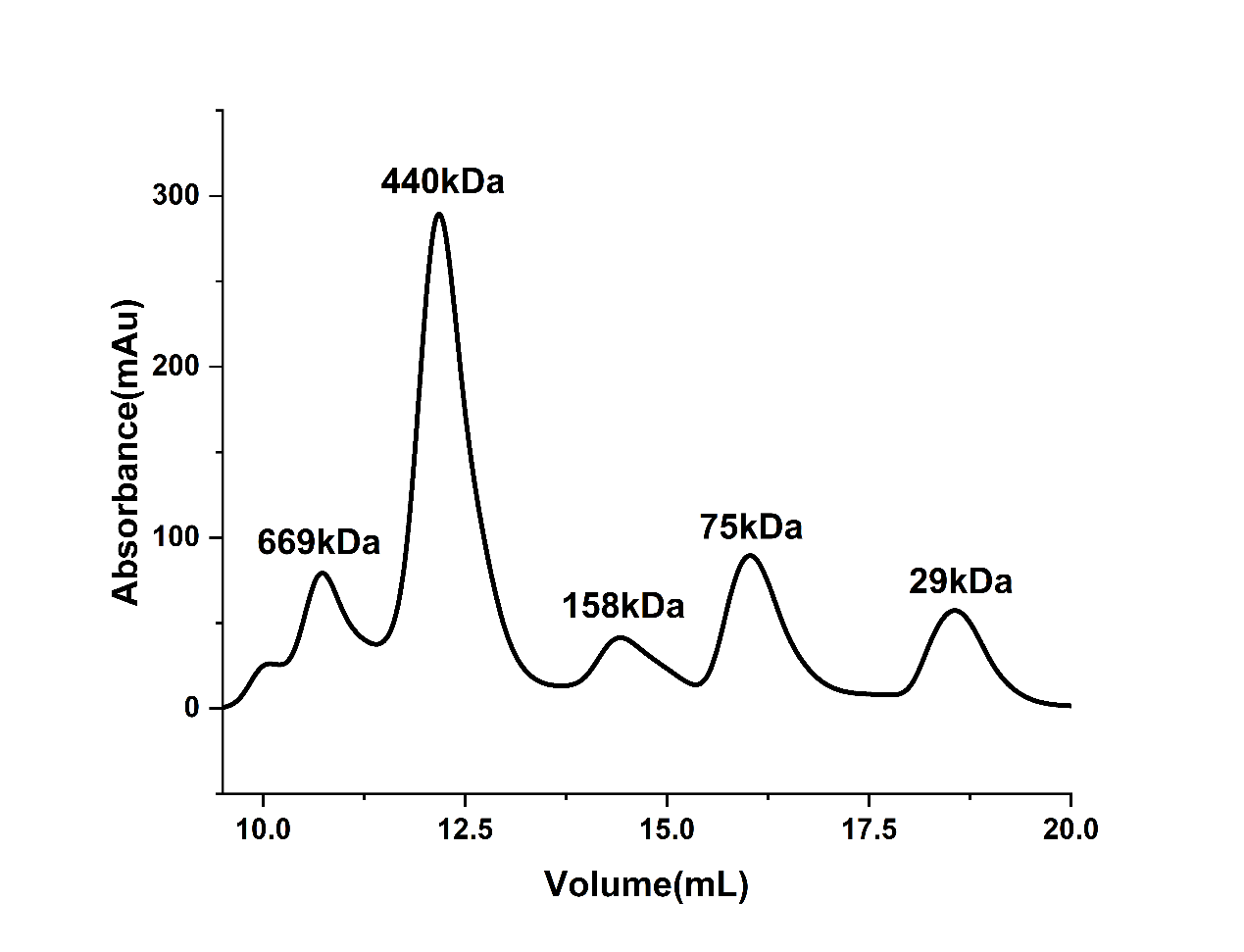


**Figure S11.** The standard curve of Superdex increase GL 10/300 gel filtration column for determination of the molecular mass and structures of the proteins in this study. The molecular weight of marker proteins used for gel filtration:669, 440, 158, 75, 29 kDa.

**
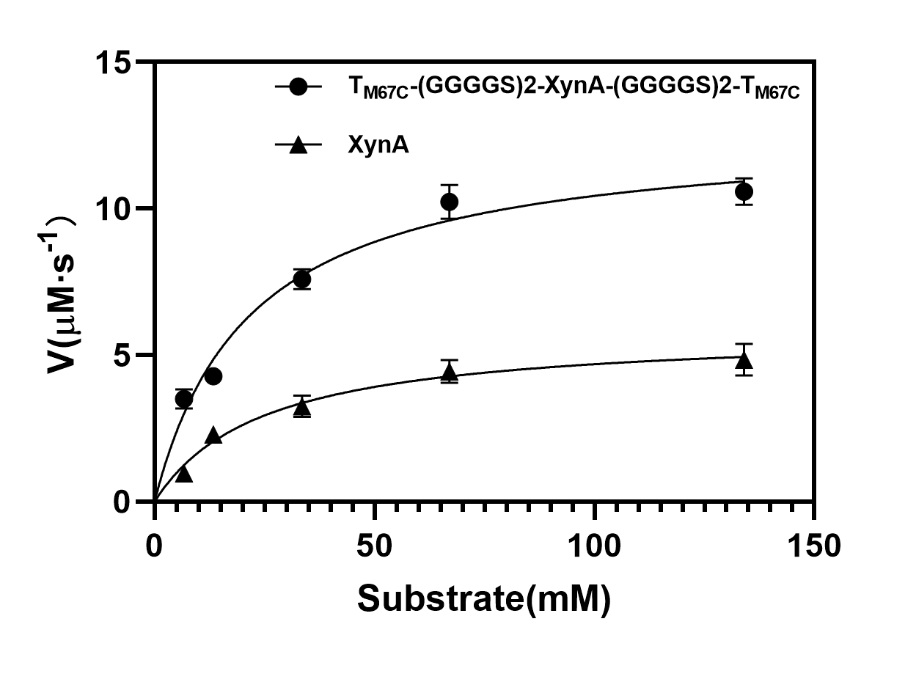
**

**Figure S12.** The plot of the kinetic constants of XynA and T_M67C_-_(GGGGS)2_-XynA-_(GGGGS)2_-T_M67C_.


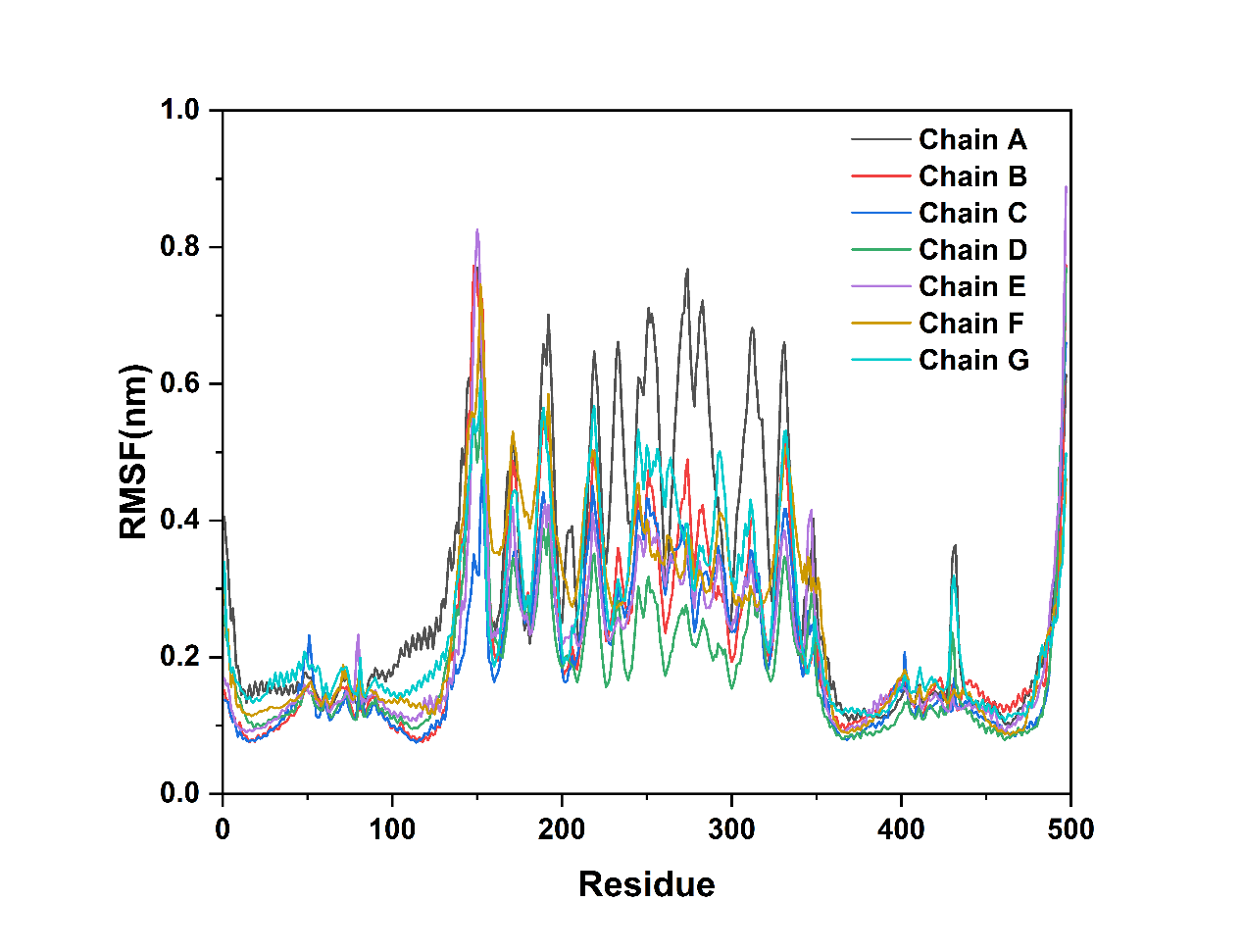


**Figure S13.** The RMSF of T_M67C_-_(GGGGS)2_-XynA-_(GGGGS)2_-T_M67C_ during MD simulation under 350K. Among them, chain D is located at the center of the multimer during the simulation process.


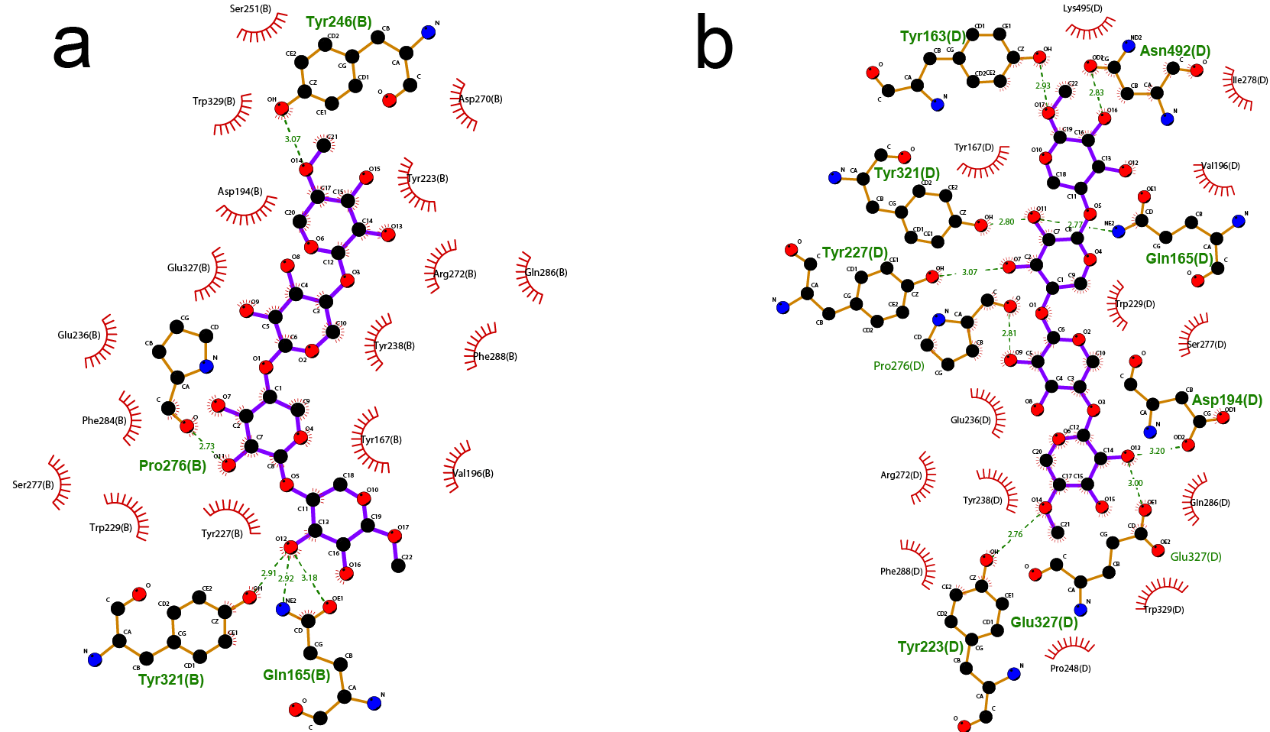


**Figure S14.** The docking of Xylan into the substrate binding pocket of a) the WT XynA XynA and b) T_M67C_-_(GGGGS)2_-XynA-_(GGGGS)2_-T_M67C._


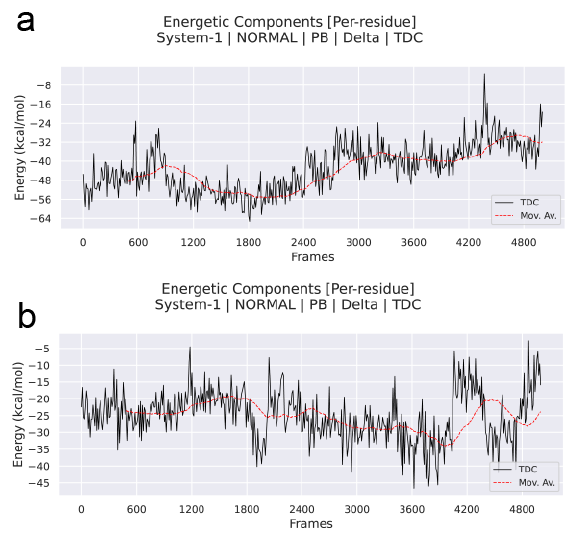


**Figure S15.** The binding free energies of XynA and T_M67C_-_(GGGGS)2_-XynA-_(GGGGS)2_-T_M67C_ with the substrate calculated using the MM/PBSA method. a) The binding free energy of XynA with the substrate. b) The binding free energy of T_M67C_-_(GGGGS)2_-XynA-_(GGGGS)2_-T_M67C_ with the substrate.

**
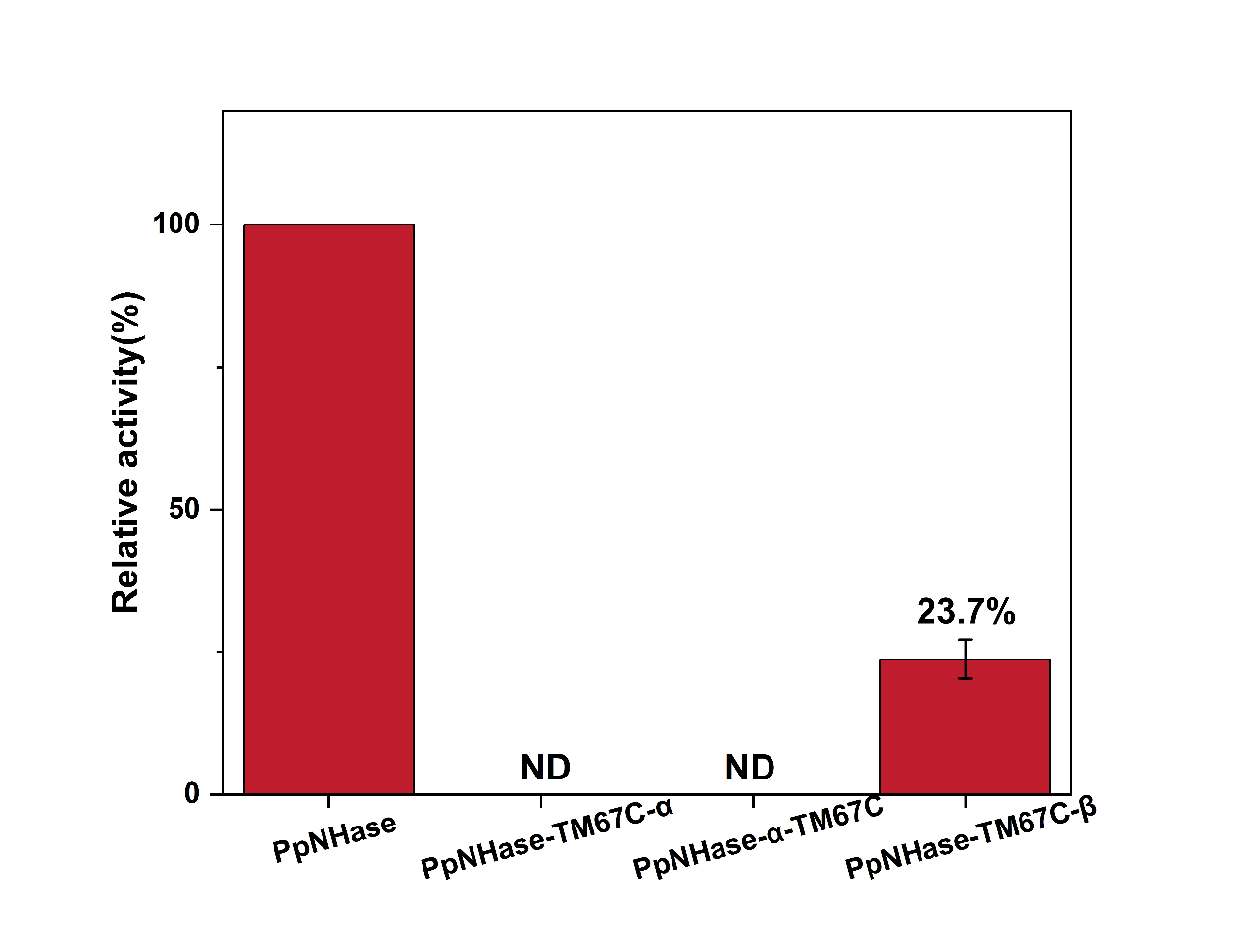
**

**Figure S16.** The relative activity of *Pp*NHase after fusion with TERM-M67C in different terminus of either α or β subunit.

**
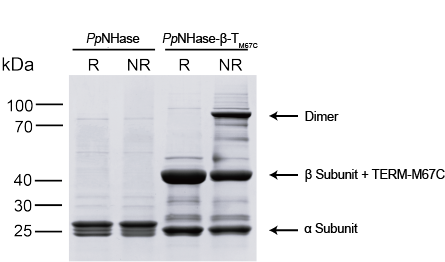
**

**Figure S17.** The SDS-PAGE analysis of the WT *Pp*NHase and *Pp*NHase-β-T_M67C_ under reducing (R) and non-reducing (NR) conditions. The molecular weight of the protein with inter-subunit disulfide bonds is approximately 80 kDa. 2-Mercaptoethanol was used as the reducing agent.

**
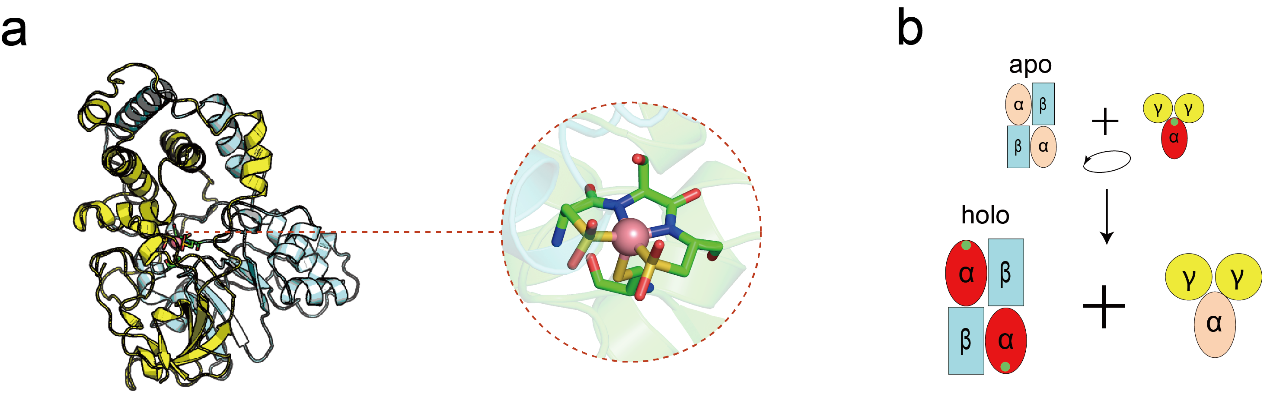
**

**Figure S18.** Self-subunit swapping for cobalt incorporation into the tetrameric NHase.


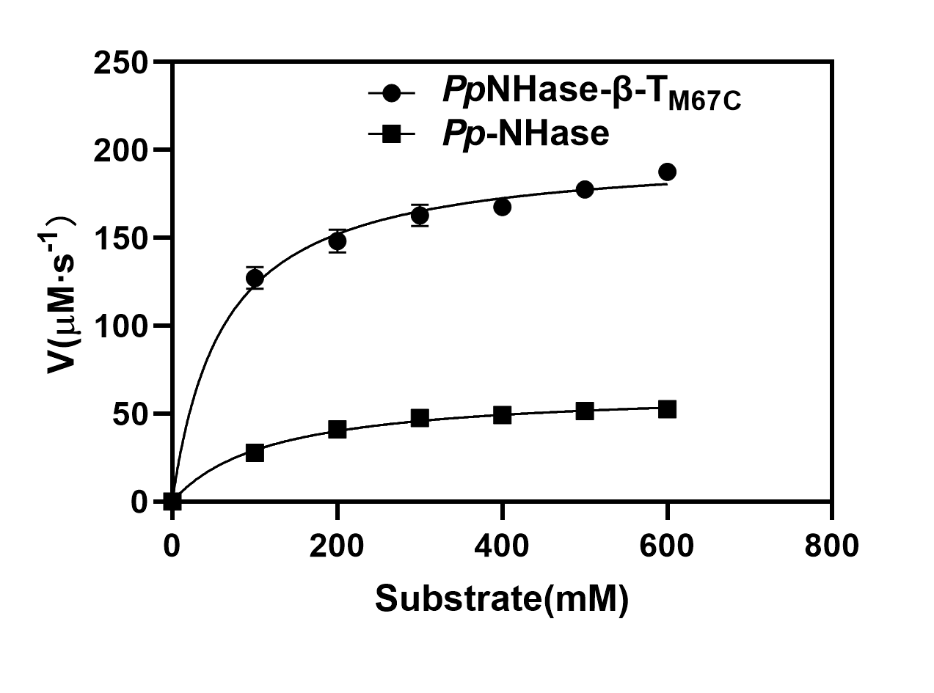


**Figure S19.** The plot of the kinetic constants of *Pp*NHase and *Pp*NHase-β-T_M67C_.


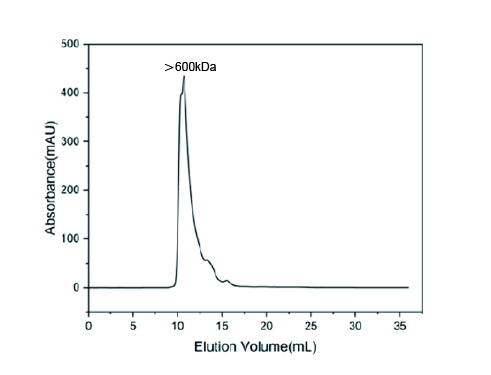


**Figure S20.** Size Exclusion Chromatography (SEC) analysis of *Pp*NHase-β-T_M67C_. When the elution volume is 10 mL, the elution peak corresponds to a molecular weight greater than 600 kDa. Superdex increase GL 10/300 gel filtration column was used.

**
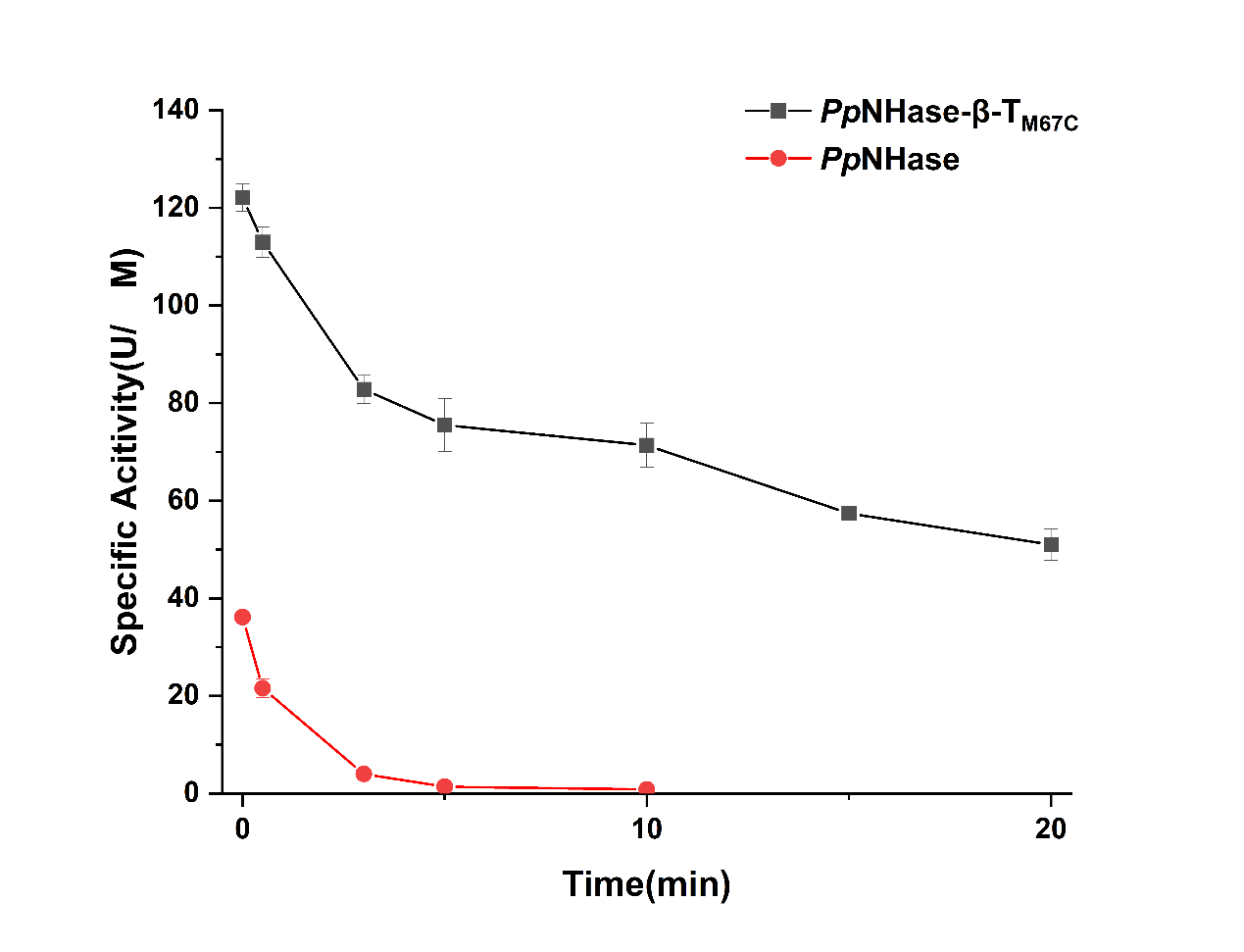
**

**Figure S21.** The half-life of *Pp*NHase and *Pp*NHase-β-T_M67C_ at 65°C. Thermal stability is characterized by statistics and analysis of relative activity.


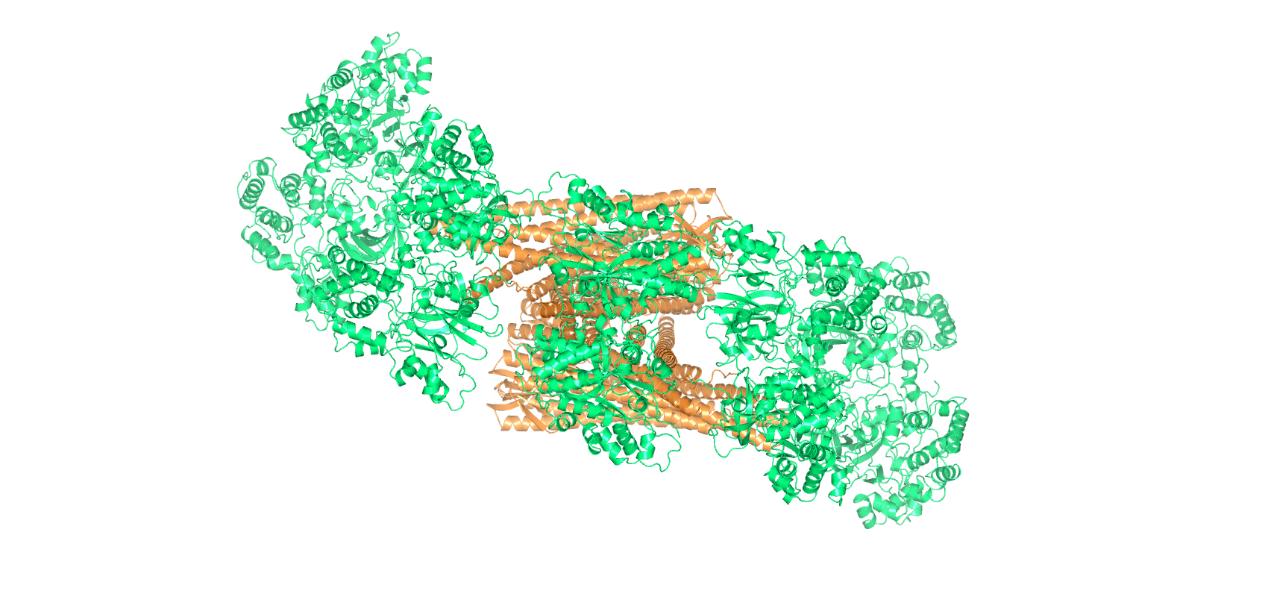

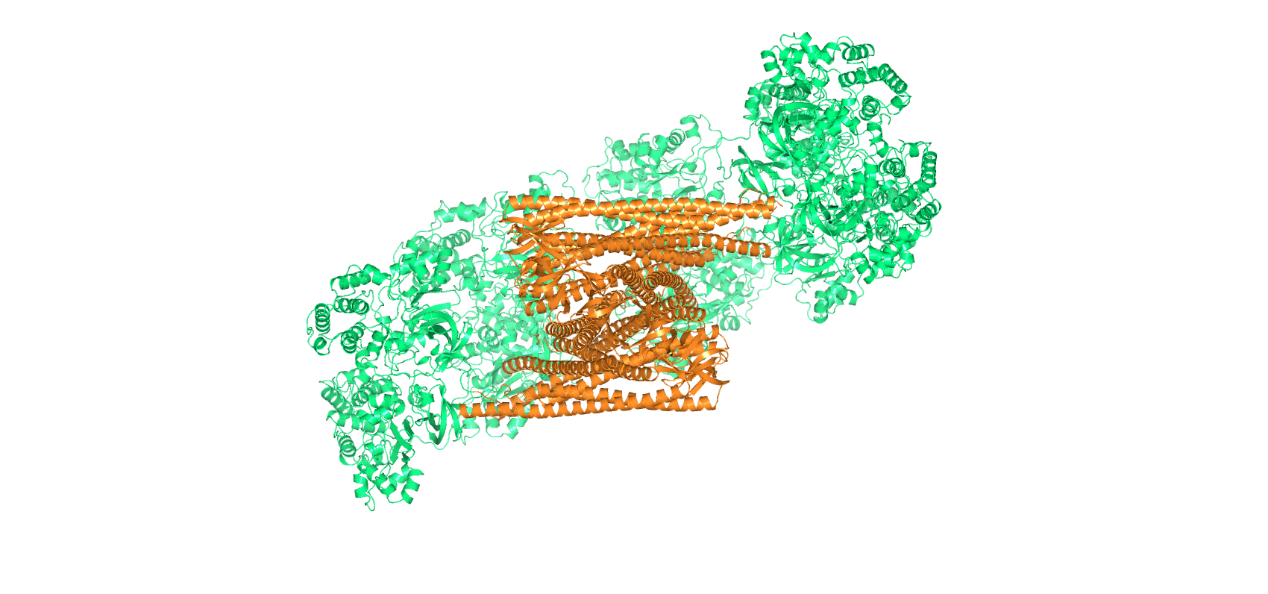


**Figure S22.** The pentameric model of *Pp*NHase-β-T_M67C_ features TERM-M67C highlighted in orange, while *Pp*NHase is shown in green.


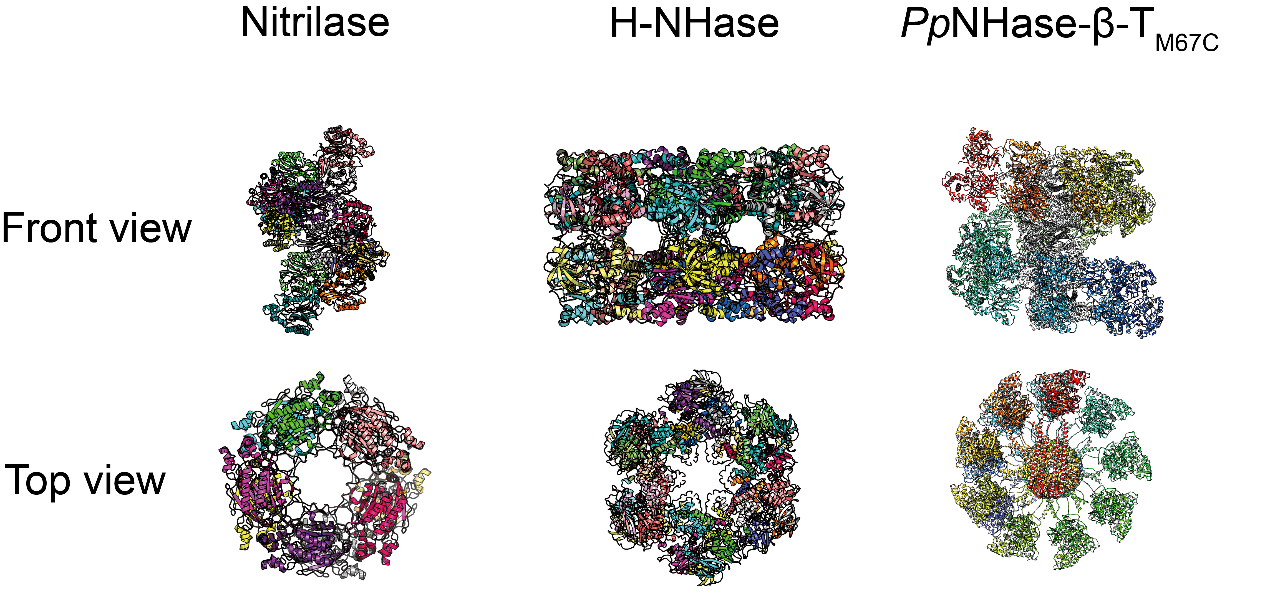


**Figure S23.** The hierarchical structure of Nitrilase (PDB ID: 8UXU). The structure of H-NHase was created by AlphaFold3. *Pp*NHase-β-T_M67C_ structure was created by AlphaFold3 and LZerD Webserver.

**
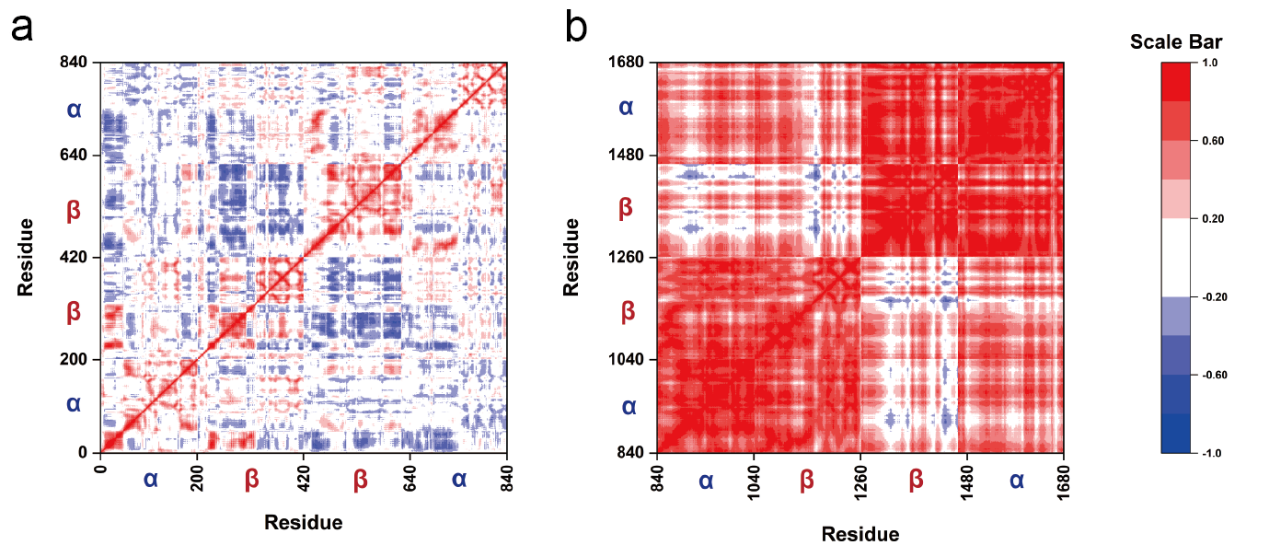
**

**Figure S24.** The DCCM of *Pp*NHase and *Pp*NHase-β-T_M67C_ at 350K. a) *Pp*NHase. b) *Pp*NHase-β-T_M67C_.


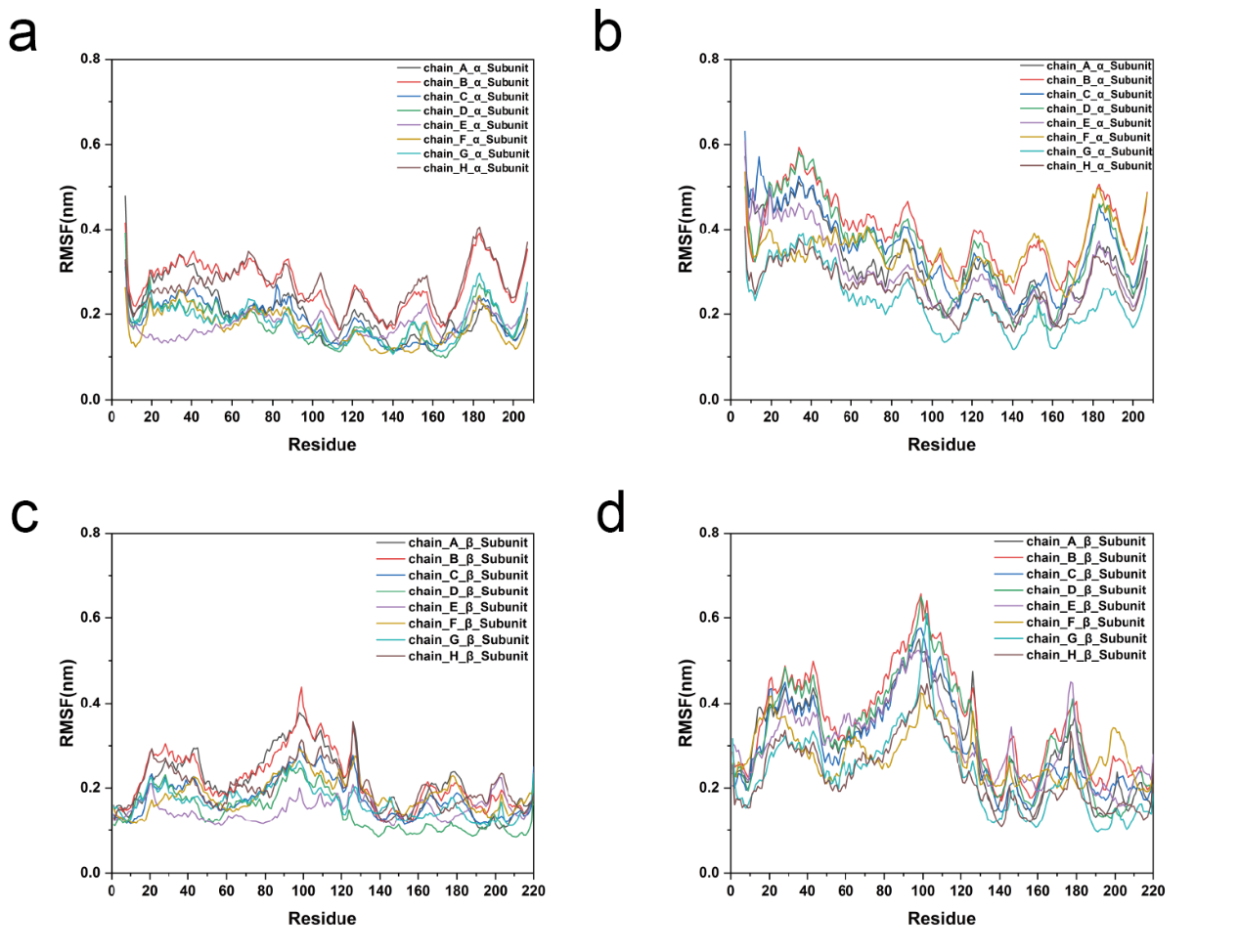


**Figure S25.** RMSF of *Pp*NHase and *Pp*NHase-β-T_M67C_’s α subunit under a) 300K. b) 350K. RMSF of *Pp*NHase-β-T_M67C_’s β subunit under c) 300K. d) 350K.


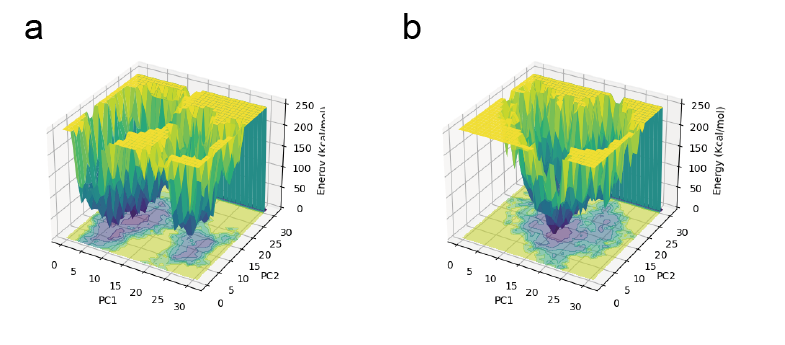


**Figure S26.** The free energy landscape of *Pp*NHase and *Pp*NHase-β-T_M67C_ at 350K. a) Two lowest free energy wells, which usually indicates the system may have two different stable states or conformations. b) Only one distinct lowest free energy well is shown, it typically indicates the system has one stable state or conformation.

**
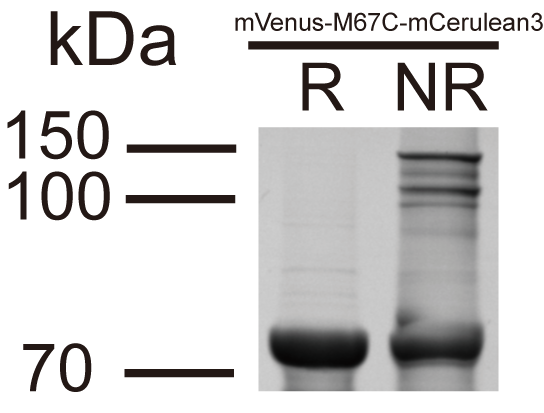

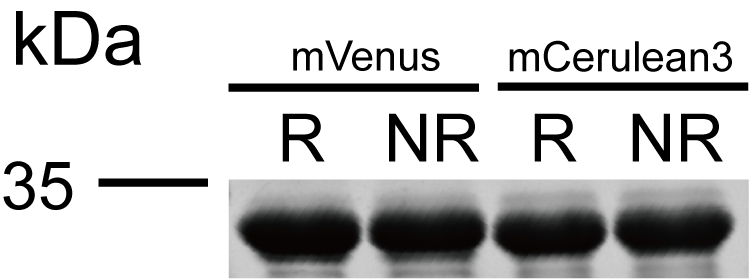
**

**Figure S27.** The SDS-PAGE of mVenus-T_M67C_-mCerulean3 and mVenus and mCerulean3 (R=reducing, NR=non-reducing).

**
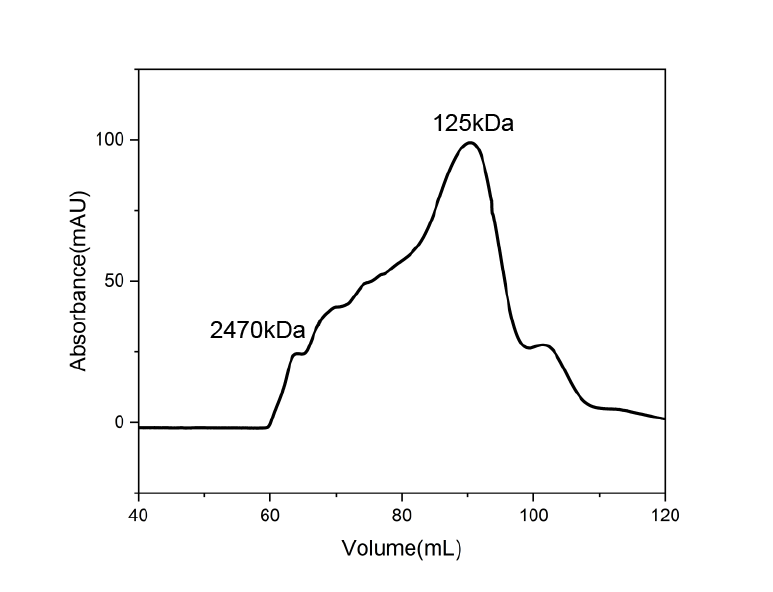
 Figure S28.** The size exclusion chromatography result of mVenus-T_M67C_-mCerulean3. The molecule weight of each peak was labeled.


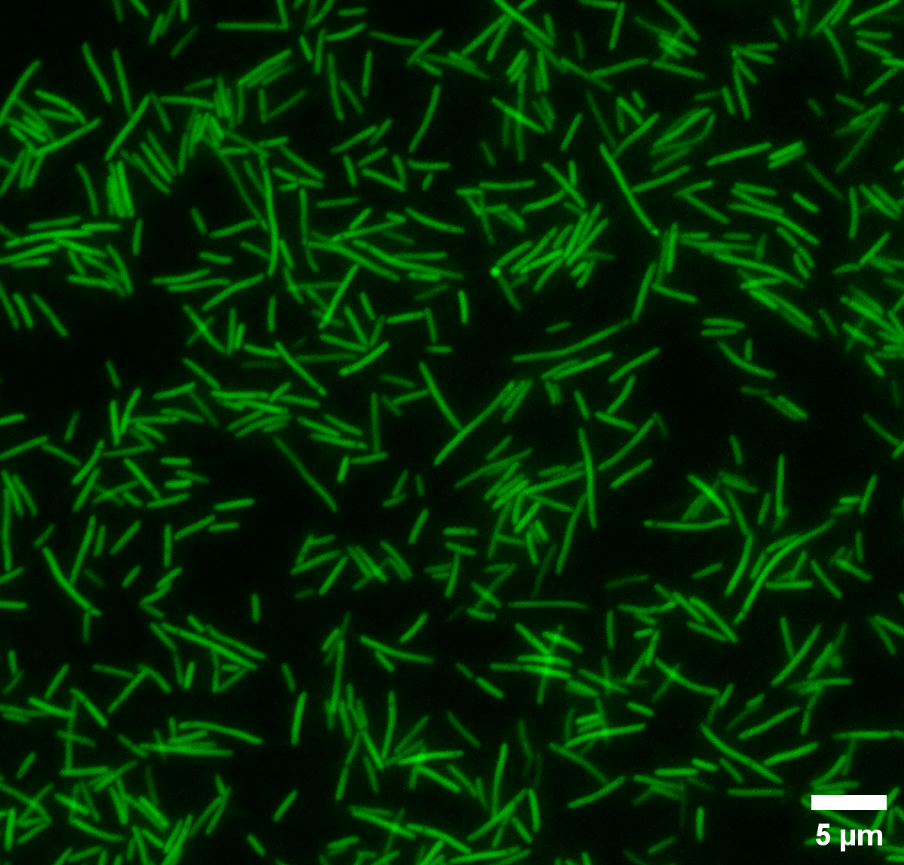


**Figure S29.** In *E. coli* expressing only the fluorescent proteins, the proteins were evenly distributed in the cytoplasm of the host cells.
